# Generalized target behavior reductions and maintenance of effects following an augmented competing stimulus assessment sequence

**DOI:** 10.1002/jaba.70021

**Published:** 2025-06-18

**Authors:** Samantha L. Breeman, Jason C. Vladescu, Tina M. Sidener, Ruth M. DeBar, Danielle Gureghian

**Affiliations:** ^1^ Children's Specialized Hospital–Rutgers University Center for Autism Research, Education, and Services (CSH–RUCARES) Somerset NJ USA; ^2^ Rutgers Brain Health Institute Rutgers University Piscataway NJ USA; ^3^ Applied Behavior Analysis Program SUNY Downstate Health Sciences University Brooklyn NY USA; ^4^ Department of Applied Behavior Analysis Caldwell University Caldwell NJ USA; ^5^ Garden Academy West Orange NJ USA

**Keywords:** automatic reinforcement, competing stimulus assessment, generalization, stereotypy subtyping

## Abstract

Competing stimulus assessments are one technology that aids in the development of treatment for automatically reinforced behavior. However, competing stimulus assessments do not always yield robust results. Stereotypic behaviors of different subtypes may require procedural modifications to successfully identify competing stimuli. The current investigation included functional analyses to determine whether participant responding aligned with proposed subtypes for such behaviors. Next, we implemented augmented competing‐stimulus‐assessment (A‐CSA) procedures across target and generalization stimuli to determine whether (a) responding across either subtype was more likely to require intensive modifications and (b) the A‐CSA procedures promoted generalized target behavior reduction within stimulus classes. Lastly, a treatment evaluation was conducted to determine the durability of these findings and the generalization of the reduced target behavior to other settings. The general applicability of the subtyping model remains unclear, but two participants demonstrated maintenance of competition effects.

Stereotypic behavior displayed by individuals receiving behavior‐analytic services often demonstrates insensitivity to social contingencies (Beavers et al., 2013; Querim et al., 2013). It is well documented that behaviors maintained by automatic reinforcement can negatively affect learning new skills (Rapp & Vollmer, 2005) and social acceptance (Welsh et al., 2019) and specific topographies can pose risk of injury (Roscoe et al., 2013). However, questions remain regarding the ethics of treating behaviors that are maintained by automatic reinforcement using procedures based on applied behavior analysis (Leaf et al., 2022). Some autistic advocates and individuals with autism spectrum disorder (ASD) disparage the treatment of stereotypic behaviors, reporting that these responses help to self‐soothe (Kapp et al., 2019) with a potential root in individuals' autonomic nervous systems (Lory et al., 2023). Also, several stereotypic behaviors, such as hair twirling, are observed among individuals of typical development (Smith & Van Houten, 1996; Woods & Miltenberger, 1996).

The Ethics Code for Behavior Analysts (Behavior Analyst Certification Board, 2020) requires behavior analysts to support client rights and act in clients' best interests, which includes deciding when to treat stereotypic behaviors. When intervention is deemed necessary by the client, their family, and the behavior analyst, treatment development, training, implementation, and continued maintenance can be resource‐intensive (Blackman et al., 2020; Brusa & Richman, 2008). Additionally, when interventions use highly‐contrived components, relapse of stereotypic behaviors may occur without consistent maintenance and high procedural fidelity (Podlesnik et al., 2017).

Moreover, the effects of interventions appear idiosyncratic. For example, researchers have attempted to match the sensory consequences of vocal stereotypy with singing (Thomas et al., 2020) and sound‐producing toys (Shawler et al., 2020). Although both interventions resulted in auditory stimulation, only the intervention described by Thomas et al. (2020) was successful. Such uncertainties could lead to the implementation of multiple unsuccessful treatments before identifying an effective program.

It may be helpful to generate a subtyping model for stereotypic behaviors similar to the model described by Hagopian et al. (2017) for subtypes of self‐injurious behaviors to help streamline treatment. Each subtype represented patterns of responding across functional analysis conditions and, as outlined by Hagopian et al., aligned with differential treatment outcomes (i.e., Subtype 1 self‐injury was less resistant to treatment than Subtypes 2 and 3). Steps for identifying and subtyping automatically maintained self‐injury are outlined in Table 1 of Hagopian et al. (2023). Wunderlich et al. (2022) conducted a retrospective analysis of the treatment literature for stereotypic behavior to extend the Hagopian et al. (2017) subtyping model to stereotypy and found that reinforcement‐based procedures may affect stereotypy in more nuanced ways than it affects self‐injury. Specifically, Hagopian et al. found that Subtype 1 self‐injury was more sensitive to reinforcement‐based procedures than were Subtypes 2 and 3, but Wunderlich et al. did not find this to be the case when the same subtyping rules were applied to stereotypy. Researchers have gathered preliminary evidence suggesting that physiological data (Lory et al., 2023) and additional analysis of functional analysis data (Virues‐Ortega et al., 2022) could also help subtype and inform treatments for stereotypy. Furthermore, Hagopian et al. (2023) presented a simplified quantitative method of subtyping automatically maintained self‐injury. Thus, the usefulness of a subtyping model for stereotypic behaviors requires further investigation.

**TABLE 1:** Participant demographics.

Participant	Age	Ethnicity	Diagnoses	Communication skills
Sabir	12 years	South Asian	ASD	Vocal speech and AAC device supplement
Sawyer	10 years	White	ASD	Vocal speech and AAC device supplement
Dakota	16 years	White	ASD, microcephaly, abnormal gait, ID, feeding difficulties, club foot	AAC device

*Note*: ASD = autism spectrum disorder; ID = intellectual disability; AAC = augmentative and alternative communication.

Competing stimulus assessments (CSAs) are one tool that behavior analysts can use to inform the treatment of behavior maintained by automatic reinforcement. These pretreatment assessments are designed to identify stimuli that reduce target behavior under free‐access conditions, through either competition or substitution (Haddock & Hagopian, 2020). Thus, they are beneficial in identifying stimuli for use in noncontingent reinforcement and differential reinforcement treatments. Piazza et al. (1998) measured engagement with items relative to baseline levels of pica in a baited environment in the first described CSA and found that hypothesized matched items competed with pica more effectively than unmatched items with similar levels of engagement. Researchers typically select items with a demonstrated reduction in target behaviors to provide noncontingently during treatment, though the specific reduction criteria are sometimes unclear (Haddock & Hagopian, 2020). However, autistic individuals may require teaching to engage with items in non‐stereotypic ways. In those cases, CSAs that follow standard procedures might not yield competing stimuli (Hagopian et al., 2020).

Haddock and Hagopian (2020) recently reviewed 15 articles (including 23 participants) and found CSA applications across various topographies of challenging behavior. In their review, Haddock and Hagopian concluded that CSAs tend to have strong predictive validity (i.e., items identified in the CSA largely continued competing with target behaviors during treatment evaluations). Therefore, increasing the likelihood that CSAs identify competing stimuli for all participants could improve treatment efficacy. Haddock and Hagopian also found a lack of information about matched and unmatched stimuli definitions. Perhaps this plays a role in why, among participants with behavior maintained by automatic reinforcement, only 38% of hypothesized matched and 43% of hypothesized unmatched stimuli were classified as effective competing stimuli.

As a result of these considerations, several researchers have developed augmented CSAs (A‐CSAs). Supporting Information A includes an overview of studies that have used A‐CSAs and the augmenting procedures they included. When no competing stimuli are identified during a CSA, researchers introduce additional procedures of increasing intrusiveness (i.e., the procedures gradually provide greater assistance and require more physical contact, which may restrict the individual's free movement; Mayton et al., 2014). For example, the addition of re‐presentation (i.e., placing items into participant's hands) following 10 s without item engagement and blocking target behavior allowed Jennett et al. (2011) to identify three competing stimuli. Hagopian et al. (2020) demonstrated that physically prompting engagement and blocking target behavior proved similarly effective. Subsequent treatment evaluations of competing stimuli that used response blocking have found that these additional components are often necessary to maintain competition effects (Schmidt et al., 2021).

Leif et al. (2020) identified competing stimuli using differential reinforcement of alternative behavior (DRA) procedures for item engagement in the context of CSAs without the need for response blocking. However, the original DRA contingency evaluated by Leif et al. involved delivering a piece of food directly into the participant's mouth contingent on 10 s of continuous item engagement; this was later thinned to every 60 s. Although promising, providing reinforcement on such dense schedules is likely not feasible in most clinical settings over the long term. Additionally, DRA procedures within A‐CSAs are currently the least investigated component to be included.

Despite these promising findings, there are some noteworthy gaps in the literature. Using A‐CSAs to inform the treatment of stereotypic behaviors has been limited. Treatments targeting item engagement or other functional object manipulation may be more socially valid than other procedures, so researchers should evaluate the utility of such assessments. Relatedly, Wunderlich et al. (2022) did not evaluate the efficacy of treatments that were informed by CSAs and those that were informed by other preassessments separately. It is unclear whether this would have affected the validation of the subtyping model. Additionally, no A‐CSA sequences published to date included tests for generalized target behavior reduction or engagement with stimuli that did not acquire a history of reinforcement within the assessment. Given that augmentations often include prompting and reinforcing item engagement, it is possible that individuals may then engage with items that share characteristics with assessed items in similar ways following the A‐CSA. However, no tests of this kind have been conducted to our knowledge. Prolonged exposure to a single item can result in satiation (Hagopian et al., 2000), and stereotypic behaviors can continue uninterrupted for extended periods (Berkson, 1983); it seems beneficial to identify multiple competing stimuli as efficiently as possible. Last, treatment evaluation sessions have not included maintenance probes to assess the continued effectiveness of identified competing stimuli.

In reviewing the literature on stereotypy subtyping models and A‐CSAs, we determined that both evaluate the effects of item access on levels of stereotypy. Laureano et al. (2023) and Frank‐Crawford et al. (2023) found evidence that Subtype 1 self‐injury correlates with more competing stimuli identified during CSAs and Subtypes 2 and 3 correlate with fewer. Perhaps this would also be the case for stereotypy. Thus, the subtyping model developed by Hagopian et al. (2017) and applied to stereotypy by Wunderlich et al. (2022) may also have predictive validity for the level of intervention required to identify competing stimuli. The purposes of the current study were to (a) determine whether Subtype 1 stereotypy required fewer or less intrusive augmentations to identify competing stimuli than Subtype 2, (b) assess whether reductions in target behaviors generalized to other items of the same stimulus class as the identified competing stimuli, and (c) conduct a treatment evaluation with sessions of longer durations than the initial assessment as well as maintenance probes.

## GENERAL METHOD

### Participants

Three adolescents with ASD were recruited from a private school that provides services based on applied behavior analysis and had a history of receiving such services. Participants needed to meet the following inclusion criteria: parental consent to participate in the study and engagement in behavior suspected of being maintained by automatic reinforcement that interfered with skill acquisition and appropriate item engagement as determined by observation, interviews with caregivers and instructional staff, and review of the data for individual education goals. Additionally, when selecting participants for recruitment, the experimenters considered the following factors: history of previous attempted treatments for the behavior, overall tolerance for exposure to new stimuli, and the presence of severe challenging behavior (such as aggression or self‐injury) at high frequencies. Potential participants were not excluded for previous unsuccessful treatment. However, potential participants who demonstrated severe challenging behavior when prompted to engage with novel stimuli or at high frequencies throughout the school day were excluded. Participant assent was obtained by allowing participants to voluntarily enter the research space (i.e., no physical assistance was used). Supporting Information B provides assent information per participant. Sabir and Sawyer also provided a vocal “yes” or head nod when asked whether they were ready to participate. Assent was deemed withdrawn in the event that participants demonstrated challenging behavior in the form of self‐injury, elopement, or disruptions paired with crying, resulting in session termination. Three sessions were terminated (one for Sabir in the treatment evaluation, two for Dakota in the A‐CSA).

Table 1 shows the participants' demographic information. Supporting Information C and D provide additional details about participants' assessment information. Sabir, a boy who completed both studies, had received 6 years of behavior‐analytic services at the time of the study. He had a prescription for both an enzyme for digestive support (i.e., Creon) and an allergy medication (i.e., Xyzal). Previous treatments for motor stereotypy included general behavior management strategies and a resetting differential‐reinforcement‐of‐other‐behavior and DRA contingency via a multiple schedule with response blocking. The term “general behavior management strategies” referred to strategies such as providing frequent opportunities for choice‐making (e.g., selecting the order of academic programs, choosing reinforcers), restricting access to reinforcers in the presence of unsafe or disruptive behavior, redirection to ongoing activities as needed, providing proactive rule statements, and providing frequent praise and reinforcement for appropriate behaviors and independent responses during programming. He had used a token economy for 6 years. After a discussion with Sabir's caregivers and clinical team, we elected to intervene on his motor stereotypy because it impeded acquisition of new skills and performing self‐care skills independently and occasionally resulted in injuries.

Sawyer, a boy who only participated in Study 1, had received 8 years of behavior‐analytic services. He was prescribed Clonidine, and his motor stereotypy was addressed via general behavior management strategies. He had used a token economy for 2 years prior to the study. Following discussion with Sawyer's caregivers and clinical team, we decided to intervene on his motor stereotypy because it negatively affected his acquisition of new skills, including communication responses, and occurred at high rates across days and contexts.

Dakota, a girl who completed both studies, had received behavior‐analytic services for 9 years before the study and had used a token economy for 4 years. Her mouthing was previously addressed using general behavior management strategies and response blocking. She required some assistance with mobility but was ambulatory. We discussed Dakota's mouthing with her caregivers and clinical team and intervened due to the risks of choking or catching and spreading illness; mouthing also occurred at high rates with a variety of items, including her clothing. It is important to note that Dakota's previous treatment was only partially successful and difficult to implement with fidelity throughout the day (i.e., therapists could not successfully block all instances of mouthing daily). Functional analysis data were collected for one additional participant (data available upon request) whose participation terminated prior to completing Study 1.

### Setting and materials

The experimenters conducted functional analysis and A‐CSA sessions in either a padded room with a connected observation room or a spare educational space emptied of materials and other students that were not associated with the study. During A‐CSA sessions, an individual desk and two chairs were kept in the room with select items and timers. All sessions were recorded using the digital camera feature on iPads, and videos were stored in a password‐protected Google Drive account requiring a two‐step verification to access.

## STUDY 1: FUNCTIONAL ANALYSIS, SUBTYPING, AND A‐CSA SEQUENCE

### Design, measurement, interobserver agreement, and procedural fidelity

The functional analyses followed a pairwise comparison design. Sabir's *motor stereotypy* was defined as scratching, tapping, or running fingers on surface for longer than 2 s; waving arms out to the side or above his head for longer than 2 s; finger play in the form of touching fingertips together and rubbing for longer than 2 s; clapping items in between his hands for longer than 2 s; putting hands in water to splash water between fingers (all attempts are included); bending at the waist with his head down while walking or running; noncontextual jumping; and bending or rubbing ears for 2 s or longer. Gross and fine motor topographies often occurred simultaneously. Sawyer's *motor stereotypy* was defined as drumming fingers in front of his eyes, hand flapping, bouncing up and down either in his seat or standing up, repetitively shaking a non‐toy item without instruction to do so, or finger play in the form of tapping fingertips together or on the table for longer than 3 s. Dakota's *mouthing* was defined as placing objects past the plane of her lips. It is important to note that Dakota did not engage in mouthing of her body parts, so this behavior was not classified as self‐injury. Trained observers collected 10‐s partial‐interval data and summarized responding as the percentage of intervals with the target behavior. The number of intervals scored with occurrence was divided by the total number of intervals and multiplied by 100 to calculate this percentage.

The experimenters followed the same criteria and calculation procedures as were used by Wunderlich et al. (2022). We defined Subtype 1 as differentiated responding across no‐interaction and control conditions, specifically with a quotient score greater than or equal to 0.50 and less than a 30% overlap between conditions. We defined Subtype 2 as undifferentiated responding across no‐interaction and control conditions, demonstrated by a quotient score less than 0.50, greater than 30% overlap between conditions, or mean level of target responding greater than 75% of 10‐s intervals. Visual analysis was used in all cases to verify differentiated responding.

Trials of the A‐CSA followed a multielement design, and the introduction was staggered across participants. The experimenters defined *item engagement* as any attempt to physically manipulate the item, beyond simply touching it, that was not consistent with the target behavior that was maintained by automatic reinforcement, destructive, or harmful to self or others (Schmidt et al., 2021). Participants' engagement with items did not need to mirror functional play but could not include placing the item into their mouth (Dakota), running or jumping with items without an instruction to do so (Sabir), rubbing ears with items (Sabir), or drumming fingers on the item while holding it in front of the eyes (Sawyer). For some items, stereotypy and functional play looked topographically similar (e.g., rubbing a plush toy), so the behavior was scored as item engagement in those instances. Trained observers collected 10‐s partial‐interval data and implemented 3‐s onset and offset criteria when recording item engagement, meaning that data collectors only scored engagement during an interval when engagement occurred for a minimum of 3 s. These data were summarized as the percentage of intervals with item engagement by dividing the number of intervals with item engagement by the total number of intervals and multiplying the result by 100.

A second observer collected data for at least 33% of sessions across all procedures. Interobserver agreement was calculated by dividing the total number of intervals with agreement by the total number of intervals (agreements and disagreements) and multiplying by 100. Mean interobserver agreement in the functional analysis was 95.83% (range: 93.33%–98.33%) for Sabir, 96.39% (range: 91.67%–100%) for Sawyer, and 99.67% (range: 98.33%–100%) for Dakota. Mean agreement in the A‐CSA was 97.45% (range: 83.33%–100%) for Sabir, 98.34% (range: 91.67%–100%) for Sawyer, and 98.46% (range: 83.33%–100%) for Dakota.

Supporting Information E and F provide the checklists that were used to score procedural fidelity in at least 30% of functional analysis and A‐CSA sessions, respectively. Data collectors divided the number of correct behaviors by the total number of prescribed behaviors and multiplied the result by 100. Mean procedural fidelity in the functional analysis was 98% (range: 92%–100%) for Sabir, 91% (range: 75%–100%) for Sawyer, and 99% (range: 96%–100%) for Dakota. Mean procedural fidelity in the A‐CSA was 99.12% (range: 86.96%–100%) for Sabir's A‐CSA sequence, 99.13% (range: 80%–100%) for Sawyer's, and 100% for Dakota's.

### Material

Across participants, the stimulus classes presented in the A‐CSA remained consistent. However, we used a random‐without‐replacement procedure to select the initial items (hereafter, target stimuli) for the assessment sequence. Specifically, one target stimulus was randomly selected per participant from each stimulus class and the remaining stimuli were reserved for generalization probes. Stimulus classes were defined as item sets sharing physical properties, allowing for similar engagement across at least one sensory modality (e.g., Play‐Doh and slime are both manipulated with the hands, can be touched, and can change shape when manipulated) yet were discriminable from one another. Interviews using the *Reinforcer Assessment for Individuals with Severe Disabilities* (Fisher et al., 1996) with the caregivers and instructional staff helped to identify the items and classes that were included. Only the yoga ball was included in both the pre‐experimental paired‐stimulus preference assessment and A‐CSA stimulus classes. The first author developed the stimulus classes and defined the critical and noncritical features through observing typically‐developing children engage with the items and noting variations among products for sale at local retailers.

Within stimulus classes, the experimenters varied at least one noncritical feature across members of the class for all generalization probes. Additionally, the classes included items that provided both hypothesized and non‐hypothesized functional reinforcers as compared to the participant's target behaviors (LeBlanc et al., 2000); it is important to note that the first author made these designations based on likely potential reinforcers for target behavior from anecdotal observation. Across participants, the first author listed the potential functional reinforcers for target behavior. Supporting Information G through I review items producing the same potential functional reinforcers, defined as *hypothesis‐based items*, and the specific functional reinforcer that was identified. Supporting Information J provides more information about the stimulus classes. Items that did not produce any of the same potential functional reinforcers were defined as *non‐hypothesis‐based items*. The number of hypothesis‐based items varied from 0 to 5 target stimuli and 0 to 15 generalization stimuli. Multiple instructors and Board‐Certified Behavior Analysts (BCBAs) sorted the proposed stimuli into stimulus classes to obtain interobserver agreement. Total agreements of sorted stimuli were divided by total agreements and disagreements and multiplied by 100. Mean interobserver agreement was 89.99% (range: 83.33%–93.33%).

### Procedure

#### Functional analysis

Each session was 10 min. The experimenters wore condition‐correlated stimuli to enhance the discriminability between the no‐interaction and control conditions (Conners et al., 2000).

##### No‐interaction

In the no‐interaction condition, the experimenter and participant entered the room. The experimenter said, “I have to work. You stay here” and pretended to work on her clipboard. No programmed consequences were provided for target behaviors. Participants had only their augmentative communication devices present.

##### Control

In the control condition, the experimenter and participant entered the observation room together. The room already contained a highly‐preferred toy that had been identified through a paired‐stimulus preference assessment (Fisher et al., 1992). These were a yoga ball (Sabir), pop tubes (Sawyer), and a pop it (Dakota); items were kept consistent across sessions. The experimenter said, “Let's play!” and allowed the participant free access to the item. Praise and physical attention (if appropriate) were provided every 30 s. There were no programmed consequences for target behavior. If the participant demonstrated the target behavior at the scheduled time for attention, then the experimenter waited for a 5 s absence of the behavior before providing attention.

#### A‐CSA

Three control and test trials were conducted per target stimulus in each condition, except for the repeated free‐access condition. Participants progressed through each condition until multiple target stimuli were associated with an 80% reduction in the target behavior relative to the no‐item control trial of that respective condition or when the full A‐CSA sequence was completed (Hagopian et al., 2020). Similar to Hagopian et al. (2020), trial durations were individualized based on participants' responding during the functional analysis. To calculate trial duration, the experimenters divided 100 by the proportion of intervals during which target behavior occurred during the no‐interaction condition, divided the resulting number by 60, and rounded the result to the nearest whole number. Trials were 3 min (Sabir and Dakota) or 7 min (Sawyer) in duration. Participants could leave the room between trials, and the experimenters provided breaks following mands. Except for the re‐presentation and DRA augmentations, the procedures followed those outlined by Hagopian et al. (2020).

##### Control

No items were presented to allow for uninterrupted measures of target behavior (i.e., there were no programmed consequences for target behaviors). Note that participants had access to their augmentative communication devices and Dakota could mouth her clothes. Control trials always immediately preceded test trials during each phase of the CSA.

##### Free access

During test trials, the experimenter placed an item (presented in a random without replacement order) within arm's reach of the participant (Hagopian et al., 2020). The experimenter said, “You can play with this if you want” (Schmidt et al., 2021). There were no programmed consequences for target behaviors or item engagement. All items were assessed in this condition. Conducting free‐access trials with all items allowed for later evaluation of the potential generality of effects among items that were not exposed to the augmentations in the A‐CSA. If participants did not demonstrate an 80% reduction of the target behavior with multiple target stimuli in this condition, then the A‐CSA sequence began. If all members of one stimulus class competed during the free‐access phase, we would have removed that class from subsequent phases of the assessment, but this did not occur.

##### Re‐presentation

If the participant did not engage with the item for 10 s, the experimenter provided the reminder, “You can play with this if you want” and put the item into the participant's hand (Jennett et al., 2011). There were no programmed consequences for target behaviors or item engagement.

##### Re‐presentation and prompting

If the participant did not engage with the item for 10 s, the experimenter said, “You can play like this,” and prompted item engagement. Prompts were specific to each participant depending on their generalized imitative repertoires. For example, Sabir responded to the model prompts, whereas Dakota required physical prompting (e.g., guiding her hands to initiate engagement with the item). Prompts lasted up to 3 s or were discontinued until the next presentation if the participant resisted the prompt. There were no programmed consequences for target behaviors or item engagement.

##### Re‐presentation, prompting, and DRA

If the participant engaged with the item for 10 s consecutively (independently or following prompts), then the experimenter provided praise and functional reinforcers (Leif et al., 2020). Supporting Information K provides additional details about the concurrent‐operants reinforcer assessment that was used to identify reinforcers. Sabir earned six tokens and exchanged them for a small food item, which was placed in a cup and consumed following the trial (i.e., Sabir could earn up to three full token boards of six tokens for three pieces of food). Dakota's reinforcer assessment indicated that neither tokens nor praise functioned as reinforcers, so the trial timer paused for brief (i.e., 3–5 s) access to an iPad following 10 s of engagement. The iPad was selected based on caregiver report and verified using the concurrent‐operants reinforcer assessment. As outlined in Supporting Information L, Dakota engaged in mouthing concurrently with the iPad, so the experimenters did not include it as a potential competing stimulus. There were no programmed consequences for target behaviors.

##### Re‐presentation, prompting, DRA, and response blocking

The experimenter blocked all instances of target behavior by gently guiding the participant's hands to the desk or their lap. The experimenter maintained light contact for 3 s before withdrawing her hands.

##### Repeated free access

Procedures were identical to the free‐access condition; one series was completed once after participants completed the A‐CSA sequence.

##### Generalization probes

Generalization probes were conducted with all generalization stimuli once during the free‐access condition and following an 80% reduction of the target topography with multiple target stimuli or completion of the A‐CSA sequence if this criterion was not met. Sabir and Dakota completed generalization probes following the re‐presentation, prompting, DRA, and response‐blocking condition. Sawyer completed generalization probes following the re‐presentation and prompting condition.

### Results and discussion

**FIGURE 1:**
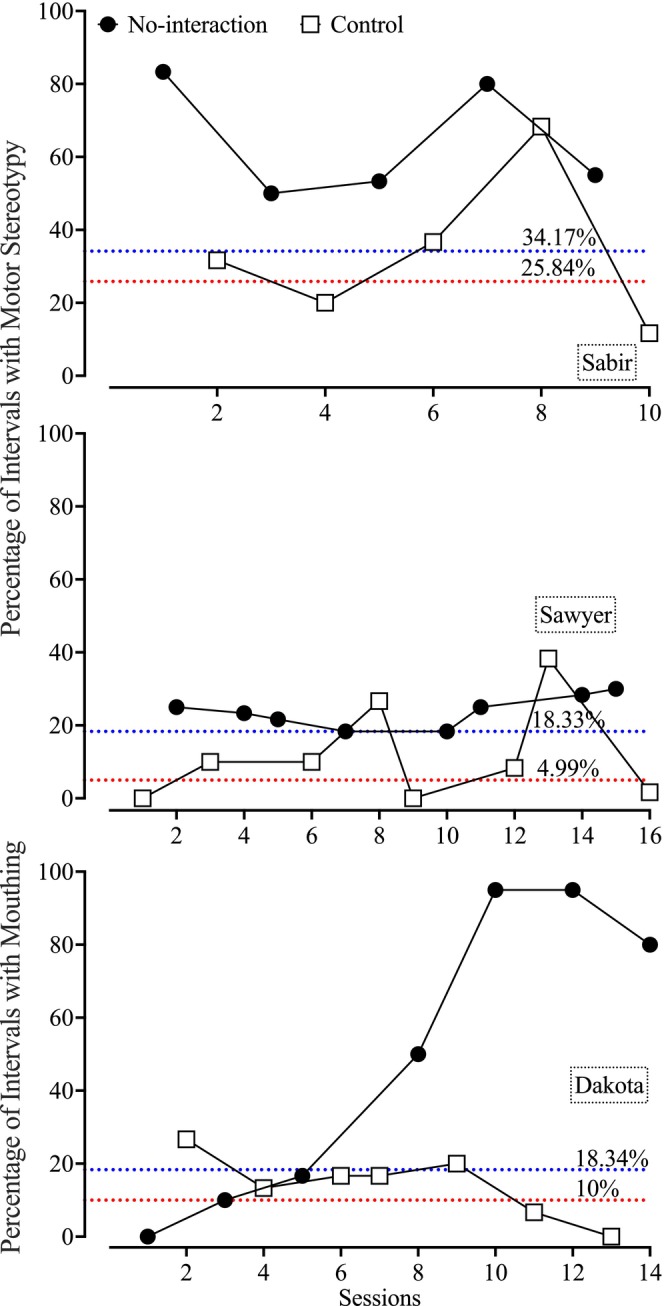
Functional analysis data. The red dotted lines represent the lower criterion lines, and the blue dotted lines represent the upper criterion lines used for subtyping.

The top panel of Figure 1 depicts Sabir's functional analysis results. With one exception, the data paths between the no‐interaction and control conditions did not overlap. Motor stereotypy occurred during a mean of 64.33% of intervals (range: 50%–83.33%) within the no‐interaction condition versus 33.67% of intervals (range: 11.67%–68.33%) in the control condition. Using the procedures from Wunderlich et al. (2022), Sabir's quotient score was 1, and his motor stereotypy met the criteria for Subtype 1.

The middle panel of Figure 1 depicts Sawyer's functional analysis results. Sawyer's responding was largely differentiated across conditions, with only two notable outliers overlapping between the control and no‐interaction conditions. He exhibited motor stereotypy during a mean of 23.75% of intervals (range: 18.33%–30%) in the no‐interaction condition and 11.88% (range: 0%–38.33%) in the control condition. Sawyer's quotient score was 0.75, and his responding was classified as Subtype 1 responding.

The bottom panel of Figure 1 depicts Dakota's functional analysis results. The no‐interaction and control data paths eventually demonstrated differentiation. Mouthing occurred during a mean of 49.52% of intervals (range: 0%–95%) in the no‐interaction condition versus a mean of 14.29% of intervals (range: 0%–26.67%) in the control condition. It is important to note that Dakota mouthed both her augmentative communication device and her clothing (i.e., strings on a sweatshirt, sleeves, hoods) during the no‐interaction condition because no other items were present. Although her quotient score was 0.43 and there was a 42.86% overlap in responding between conditions despite the eventual differentiation, a clear increasing trend is seen in the second half of her assessment. This, paired with the recommendations by Hagopian et al. (2023) to subtype behavior via level of differentiation rather than quotient scores, support a Subtype 1 categorization for Dakota's mouthing.

These data suggest that the subtyping model may have generality for stereotypic behaviors. Furthermore, given that all participants demonstrated reduced levels of target behavior in the control condition, competing stimuli might be an effective treatment. Future stereotypy subtyping may be better informed by using the simplified method proposed by Hagopian et al. (2023). Separating and subtyping topographies of stereotypy as distinct classes may have been beneficial, especially for Sabir. It remains important to assess whether such distinctions affect treatment outcomes and validate separate subtypes.

**FIGURE 2:**
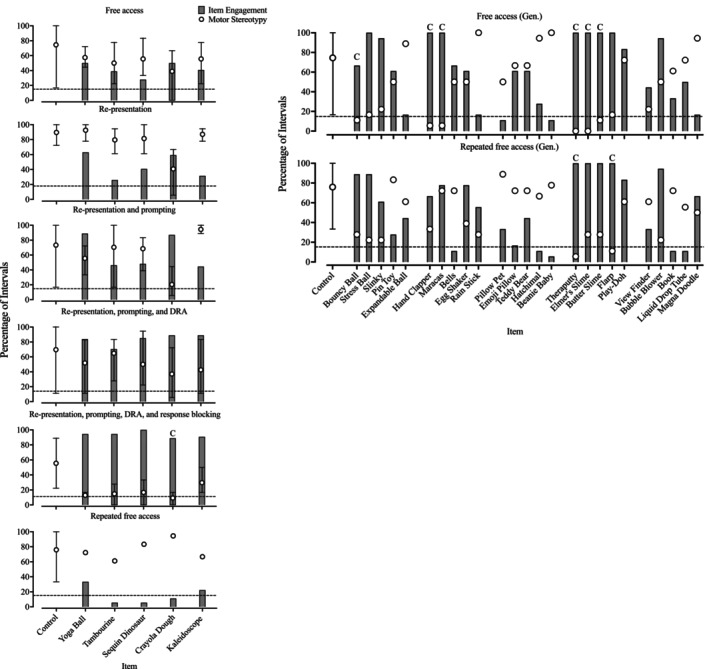
Sabir's A‐CSA sequence data. The dashed horizontal line indicates the value at which an 80% reduction would be observed. The graphed data are representative of mean percentage of intervals during which target behavior occurred, and the error bars indicate the range of raw percentage of intervals of target behavior observed across trials. The letter “C” signals that the stimulus produced an 80% reduction in target behavior. A‐CSA = augmented competing stimulus assessment; Gen = Generalization stimuli.

Figure 2 depicts Sabir's A‐CSA data. Table 2 outlines the observed percentages of reduction across stimuli. None of the target stimuli produced an 80% reduction in motor stereotypy during the free‐access condition (top left panel; *M* = 74.5%, range: 16.67%–100%); however, six generalization stimuli (top right panel) were associated with an 80% reduction in stereotypy (i.e., bouncy ball, hand clapper, maracas, Theraputty, Elmer's slime, and butter slime). Item engagement with target stimuli was low to moderate, whereas engagement with the generalization stimuli occurred in 100% of intervals, apart from the bouncy ball. Overall levels of motor stereotypy decreased slightly during control trials across the re‐presentation (second left panel; *M* = 89.58%, range: 72.22%–100%); re‐presentation and prompting (third left panel; *M* = 73.33%, range: 16.67%–100%); and re‐presentation, prompting, and DRA (fourth left panel; *M* = 69.63%, range: 11.11%–100%) conditions. Motor stereotypy persisted at moderate levels within the re‐presentation, prompting, DRA, and response‐blocking condition (fifth left panel) control trials (*M* = 55.56%, range: 22.22%–88.89%), although Crayola Dough produced an 80% reduction. Notably, item engagement with all target stimuli increased from the free‐access condition, as indicated by the gray bars in the fourth and fifth panels. Motor stereotypy increased in the repeated free‐access condition (sixth left panel) control trials (*M* = 75.93%, range: 33.33%–100%), and, unlike the previous free‐access condition, only Theraputty and Flarp (bottom right panel) produced an 80% reduction despite high item engagement with most members of Crayola Dough's stimulus class.

**TABLE 2:** Percentages of reduction in mouthing in A‐CSA and repeated free access for Sabir.

Stimulus class	Stimulus	Free access		Response blocking		Repeated Free access	
		% Intervals	% Reduction	% Intervals	% Reduction	% Intervals	% Reduction
Malleable shape toys	Crayola Dough	38.89 (*M*)	47.79 (*M*)	9.26 (*M*)	83.33 (*M*)^a^	94.44	−24.38
	Flarp	16.67	77.62			11.11	85.37^a^
	Elmer's Slime	0	100^a^			27.78	63.41
	Theraputty	0	100^a^			5.56	92.68^a^
	Butter Slime	11.11	85.09^a^			27.78	63.41
	Play‐Doh	72.22	3.06			61.11	19.52
Plush touch toys	Sequin Dinosaur	55.56 (*M*)	25.42 (*M*)	16.67 (*M*)	69.99 (*M*)	83.33	−9.75
	Emoji Pillow	66.67	10.51			72.22	4.89
	Pillow Pet	50	32.89			88.89	−17.07
	Hatchimal	94.44	−26.77			66.67	12.19
	Teddy Bear	66.67	10.51			72.22	4.89
	Beanie Baby	100	−34.23			77.78	−2.44
Visual effects toys	Kaleidoscope	55.56 (*M*)	25.42 (*M*)	29.63 (*M*)	46.67 (*M*)	66.67	12.19
	Liquid Drop Tube	72.22	3.06			55.56	26.83
	Bubble Blower	50	32.89			22.22	70.74
	Magna Doodle	94.44	−26.77			50	34.15
	View Finder	22.22	70.17			61.11	19.52
	Book	61.11	17.97			72.22	4.89
Kinesthetic tactile toys	Yoga Ball	57.40 (*M*)	22.95 (*M*)	12.96 (*M*)	76.67 (*M*)	72.22	4.89
	Expandable Ball	88.89	−19.32			61.11	19.52
	Pin Toy	50	32.89			83.33	−9.75
	Slinky	22.22	70.17			22.22	70.74
	Stress Ball	16.67	77.62			22.22	70.74
	Bouncy Ball	11.11	85.09^a^			27.78	63.41
Shaken noise maker toys	Tambourine	49.99 (*M*)	32.89 (*M*)	14.82 (*M*)	73.33 (*M*)	61.11	19.52
	Egg Shaker	50	32.89			38.89	48.78
	Hand Clapper	5.56	92.54^a^			33.33	56.10
	Bells	50	32.89			72.22	4.89
	Rain Stick	100	−34.23			27.78	63.41
	Maracas	5.56	92.54^a^			72.22	4.89

*Note*: Percentages of reduction were calculated using the mean percentage of responding during the control trials for that phase of the A‐CSA sequence. A‐CSA = augmented competing stimulus assessment; (*M*) indicates that the value represents the mean percentage (i.e., the value was derived from multiple trials).

^a^ Indicates that the stimulus was associated with an 80% reduction in the target behavior.

**FIGURE 3:**
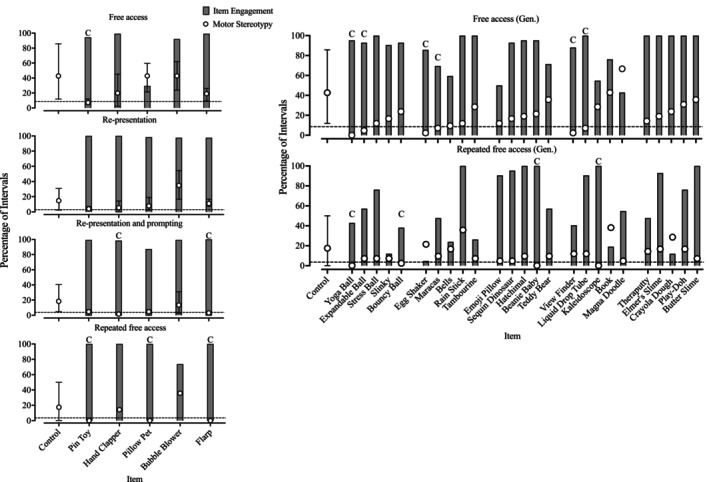
Sawyer's A‐CSA sequence data. The dashed horizontal line indicates the value at which an 80% reduction would be observed. The graphed data are representative of mean percentage of intervals during which target behavior occurred, and the error bars indicate the range of raw percentage of intervals of target behavior observed across trials. The letter “C” signals that the stimulus produced an 80% reduction in target behavior. A‐CSA = augmented competing stimulus assessment; Gen = Generalization stimuli.

Figure 3 depicts Sawyer's responding throughout the A‐CSA, and Table 3 outlines the observed percentages of reduction. Within the free‐access condition (top left panel), one target stimulus (i.e., the pin toy) produced an 80% reduction in motor stereotypy and item engagement (as indicated by the gray bars) was high with all items except for the Pillow Pet. Additionally, several generalization stimuli (top right panel) produced similar reductions (i.e., yoga ball, expandable ball, egg shaker, maracas, View Finder, and liquid drop tube) and item engagement was moderate to high. Upon implementing the re‐presentation condition (second panel on the left), Sawyer's mean motor stereotypy decreased substantially in control trials (*M* = 14.76%, range: 2.38%–30.95%) because he began demonstrating an incompatible behavior (i.e., nose plugging). During the re‐presentation and prompting condition (third panel on the left), mean motor stereotypy in control trials remained low (*M* = 18.41%, range: 4.76%–40.48%). Despite this, the hand clapper and Flarp produced an 80% reduction in motor stereotypy and two additional stimuli (i.e., pin toy and Pillow Pet) produced a 74% reduction in motor stereotypy; Sawyer engaged with all target stimuli for the majority of intervals each trial. In the repeated free‐access condition (bottom left panel), Sawyer's mean motor stereotypy in the control trials remained similar to that observed in the previous conditions (*M* = 17.46%, range: 0%–50%) but three target stimuli (i.e., pin toy, Pillow Pet, and Flarp) produced an 80% reduction and were associated with high levels of engagement. During the generalization trials within the repeated free‐access condition (bottom right panel), four generalization stimuli (i.e., yoga ball, bouncy ball, Beanie Baby, and kaleidoscope) produced an 80% reduction but engagement with these items was variable (i.e., engagement with the yoga ball and bouncy ball were much lower than with the Beanie Baby and kaleidoscope). Of these generalization stimuli, both the yoga ball and bouncy ball were from the same stimulus class as the pin toy, and the Beanie Baby was from the same class as the Pillow Pet. That similar reductions in motor stereotypy were observed with the kaleidoscope, which was not in the same class as any of the target stimuli that produced an 80% reduction, poses questions about the mechanism responsible for these results.

**TABLE 3:** Percentages of reduction in motor stereotypy in A‐CSA and repeated free access for Sawyer.

Stimulus class	Stimulus	Free access		Prompting		Repeated free access	
		% Intervals	% Reduction	% Intervals	% Reduction	% Intervals	% Reduction
Malleable shape toys	Flarp	19.05 (*M*)	55.43 (*M*)	3.17 (*M*)	82.78 (*M*)^a^	0	100^a^
	Crayola Dough	23.81	44.29			28.57	−63.63
	Elmer's Slime	19.05	55.43			16.67	4.52
	Theraputty	14.29	66.57			14.29	18.16
	Butter Slime	35.71	16.45			7.14	59.11
	Play‐Doh	30.95	27.59			16.67	4.52
Plush touch toys	Pillow Pet	42.86 (*M*)	−0.28 (*M*)	4.76 (*M*)	74.14 (*M*)	0	100^a^
	Emoji Pillow	11.90	72.16			4.76	72.74
	Sequin Dinosaur	16.67	60.99			4.76	72.74
	Hatchimal	19.05	55.43			9.52	45.48
	Teddy Bear	35.71	16.45			9.52	45.48
	Beanie Baby	21.43	49.86			0	100^a^
Visual effects toys	Bubble Blower	42.86 (*M*)	−0.28 (*M*)	13.49 (*M*)	26.72 (*M*)	35.71	−104.52
	Liquid Drop Tube	7.14	83.29^a^			11.90	31.84
	Kaleidoscope	28.57	33.15			0	100^a^
	Magna Doodle	66.67	−55.99			4.76	72.74
	View Finder	2.38	94.43^a^			11.90	31.84
	Book	42.86	−0.28			38.09	−118.16
Kinesthetic tactile toys	Pin Toy	7.14 (*M*)	83.29 (*M*)^a^	4.76 (*M*)	74.14 (*M*)	0	100^a^
	Expandable Ball	4.76	88.86^a^			7.14	59.11
	Yoga Ball	0	100^a^			0	100^a^
	Slinky	16.67	60.99			7.14	59.11
	Stress Ball	11.90	72.16			7.14	59.11
	Bouncy Ball	23.81	44.29			2.38	86.37^a^
Shaken noise maker toys	Hand Clapper	19.84 (*M*)	53.58 (*M*)	1.59 (*M*)	91.36 (*M*)^a^	14.29	18.16
	Egg Shaker	2.38	94.43^a^			21.43	−22.74
	Tambourine	28.57	33.16			7.14	59.11
	Bells	9.52	77.73			16.67	4.52
	Rain Stick	11.90	72.16			35.71	−104.52
	Maracas	7.14	83.29^a^			9.52	45.48

*Note*: Percentages of reduction were calculated using the mean percentage of responding during the control trials for that phase of the A‐CSA sequence. A‐CSA = augmented competing stimulus assessment; (*M*) indicates that the value represents the mean percentage (i.e., the value was derived from multiple trials).

^a^ Indicates that the stimulus was associated with an 80% reduction in the target behavior.

**FIGURE 4:**
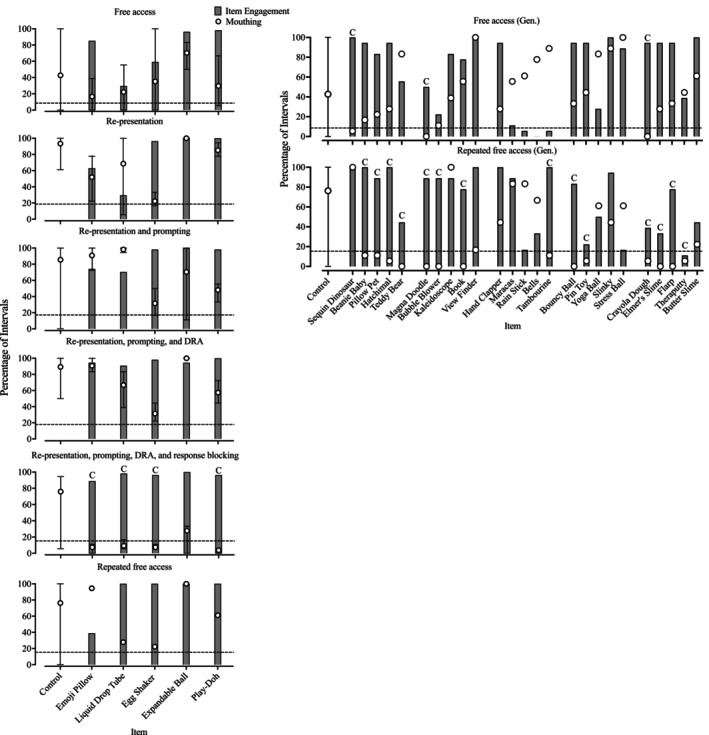
Dakota's A‐CSA sequence data. The dashed horizontal line indicates the value at which an 80% reduction would be observed. The graphed data are representative of mean percentage of intervals during which target behavior occurred, and the error bars indicate the range of raw percentage of intervals of target behavior observed across trials. The letter “C” signals that the stimulus produced an 80% reduction in target behavior. A‐CSA = augmented competing stimulus assessment; Gen = Generalization stimuli.

Figure 4 depicts Dakota's responding in the A‐CSA, and Table 4 presents the observed percentages of reduction. During the free‐access condition (top left panel), Dakota engaged in mouthing during a moderate proportion of intervals in control trials (*M* = 42.72%, range: 0%–100%). None of the target stimuli produced an 80% reduction in mouthing, but three generalization stimuli (top right panel, i.e., sequin dinosaur, Magna Doodle, and Crayola Dough) did. In comparing the top left and right panels, her engagement with target and generalization stimuli from the same classes was similar despite the differing levels of mouthing behavior observed. Within the control trials, Dakota continued to engage in elevated levels of mouthing in the re‐presentation (second left panel; *M* = 93.33%, range: 61.11%–100%); re‐presentation and prompting (third left panel; *M* = 85.55%, range: 0%–100%); and re‐presentation, prompting, and DRA (fourth left panel; *M* = 89.26%, range: 50%–100%) condition control trials. When implementing the re‐presentation, prompting, DRA, and response‐blocking conditions (fifth left panel), mouthing continued at moderate levels in the control trials (*M* = 75.93%, range: 5.56%–94.44%) but four stimuli competed (i.e., emoji pillow, liquid drop tube, egg shaker, and Play‐Doh). Item engagement with two of these (i.e., the liquid drop tube and egg shaker) demonstrated increases from the free‐access condition (comparing the top and fifth left panels), and the remaining two produced fairly stable engagement throughout the assessment to this point. In the repeated free‐access condition (sixth left panel), mouthing during the control trials remained high (*M* = 76.29%, range: 0%–100%), but 14 of the generalization stimuli (bottom right panel) produced an 80% reduction. Twelve of the generalization stimuli were from the same stimulus classes as the target stimuli that produced an 80% reduction in the previous condition. Dakota's item engagement with four of the target stimuli (i.e., liquid drop tube, egg shaker, expandable ball, and Play‐Doh) remained high despite an increase in mouthing during those trials; three of the generalization stimuli identified in this condition (i.e., Magna Doodle, bubble blower, and tambourine) occasioned greater item engagement than the free‐access condition as well.

**TABLE 4:** Percentages of reduction in motor stereotypy in A‐CSA and repeated free access for Dakota.

Stimulus class	Stimulus	Free access		Response blocking		Repeated free access	
		% Intervals	% Reduction	% Intervals	% Reduction	% Intervals	% Reduction
Malleable shape toys	Play‐Doh	29.63 (*M*)	30.64 (*M*)	3.71 (*M*)	95.11 (*M*)^a^	61.11	19.89
	Crayola Dough	0	100^a^			5.56	92.71^a^
	Flarp	33.33	21.98			0	100^a^
	Elmer's Slime	27.78	34.97			0	100^a^
	Theraputty	44.44	−4.03			5.56	92.71^a^
	Butter Slime	61.11	−43.05			22.22	70.87
Plush touch toys	Emoji Pillow	16.67 (*M*)	60.98 (*M*)	7.41 (*M*)	90.24 (*M*)^a^	94.44	−23.79
	Sequin Dinosaur	5.56	86.99^a^			100	−31.08
	Pillow Pet	22.22	47.99			11.11	85.44^a^
	Hatchimal	27.78	34.97			5.56	92.71^a^
	Teddy Bear	83.33	−95.06			0	100^a^
	Beanie Baby	16.67	60.98			11.11	85.44^a^
Visual effects toys	Liquid Drop Tube	22.22 (*M*)	47.99 (*M*)	9.26 (*M*)	87.80 (*M*)^a^	27.78	63.59
	Bubble Blower	11.11	73.99			0	100^a^
	Kaleidoscope	38.89	8.97			100	−31.08
	Magna Doodle	0	100^a^			0	100^a^
	View Finder	100	−134.08			16.67	78.15
	Book	55.56	−30.06			0	100^a^
Kinesthetic tactile toys	Expandable Ball	70.37 (*M*)	−64.72 (*M*)	27.75 (*M*)	63.45 (*M*)	100	−31.08
	Yoga Ball	83.33	−95.06			61.11	19.89
	Pin Toy	44.44	−4.03			5.56	92.71^a^
	Slinky	88.89	−108.08			44.44	41.75
	Stress Ball	100	−134.08			61.11	19.89
	Bouncy Ball	33.33	21.98			0	100^a^
Shaken noise maker toys	Egg Shaker	35.18 (*M*)	17.65 (*M*)	7.41 (*M*)	90.24 (*M*)^a^	22.22	70.87
	Tambourine	88.89	−108.08			11.11	85.44^a^
	Hand Clapper	27.78	34.97			44.44	41.75
	Bells	77.78	−82.07			66.67	12.61
	Rain Stick	61.11	−43.05			83.33	−9.23
	Maracas	55.56	−30.06			83.33	−9.23

*Note*: Percentages of reduction were calculated using the mean percentage of responding during the control trials for that phase of the A‐CSA sequence. A‐CSA = augmented competing stimulus assessment; (*M*) indicates that the value represents the mean percentage (i.e., the value was derived from multiple trials).

^a^ Indicates that the stimulus was associated with an 80% reduction in the target behavior.

During the free‐access condition, six (Sabir), seven (Sawyer), and three (Dakota) total competing stimuli were identified. Response blocking was necessary to identify multiple competing target stimuli for two participants, leading to two (Sabir), seven (Sawyer), and 14 (Dakota) total competing stimuli identified in the repeated free‐access condition. The reason for Sabir's decrease in identified stimuli is unclear when similar procedures produced a sizeable increase in Dakota's identified stimuli. As Hagopian et al. (2020) noted, response‐blocking procedures preclude us from saying that a stimulus competed independently of experimenter intervention; however, blocking may have allowed other contingencies to shift responding away from target behaviors.

Upon completing the A‐CSA, it remained to be seen whether identified competing target and generalization stimuli continued competing in more naturalistic settings during longer sessions. Study 2 was designed to evaluate the durability of these findings using two target and generalization stimuli for both Sabir and Dakota (i.e., four identified competing stimuli each).

## STUDY 2: TREATMENT EVALUATION

### Design, measurement, interobserver agreement, and procedural fidelity

Study 2 included a combined reversal and multielement design. Trained observers collected 10‐s partial‐interval data on item engagement for items identified in the A‐CSA and a moderately preferred item, as well as target topographies of behavior maintained by automatic reinforcement using previously developed definitions. We summarized these data as percentage of intervals with item engagement and target behavior, respectively; we calculated this percentage by dividing the number of intervals with each behavior by the total number of intervals and multiplying the result by 100. A second observer collected data for at least 33% of all treatment evaluation sessions across all conditions. Interobserver agreement was calculated by dividing the number of intervals with agreement by the total number of intervals and multiplying by 100. The mean interobserver agreement for Sabir was 98.10% (range: 90.83%–100%) and 99.35% (range: 97.5%–100%) for Dakota.

Supporting Information M provides the checklist that trained observers used for collecting procedural fidelity data for at least 30% of treatment evaluation sessions. Data collectors divided the number of behaviors correctly completed by the total number of expected behaviors and multiplied the result by 100 to obtain a procedural fidelity score for that session. Mean procedural fidelity was 99.35% (range: 96%–100%) for Sabir and 99.24% (range: 91.67%–100%) for Dakota.

### General procedure

All sessions were 10 min in duration and were conducted in participants' primary classrooms while other staff and students unrelated to the study were present. The term “competing stimulus continued evaluation sessions” refers to sessions where participants experienced repeated exposure to competing stimuli for relatively longer durations than during the A‐CSA. Experimenters presented the two target stimuli that produced the largest reductions in target behavior and two generalization stimuli from those same classes that also produced large reductions. In the case of multiple stimuli producing the same percentage of reduction, the research team selected the item with greater item engagement and clinical utility (e.g., incompatible with other target behaviors). Sabir used yoga ball and Crayola Dough (target stimuli) and slinky and Theraputty (generalization stimuli). Sabir demonstrated greater item engagement with the bouncy ball and stress ball than the slinky (bottom‐right panel in Figure 2), but he began picking them apart and inserting the pieces into his mouth, so we used the slinky. Dakota's target stimuli were egg shaker and Play‐Doh, and her generalization stimuli were tambourine and Flarp. A moderately preferred item, selected during at least 70% of trials during the preference assessment prior to the functional analysis, was also available for all sessions and withheld throughout the rest of participants' school day. Moderately‐preferred items were present throughout all sessions to improve the social validity of the 10‐min baseline sessions within the school setting (i.e., stakeholders deemed it inappropriate for participants to sit in the absence of an activity). Sabir's moderately preferred item was a sensory bean bag and Dakota's was a plush doll. As such, the moderately preferred items shared some properties with the competing stimuli (e.g., produced tactile stimulation) but differed in several ways. The potential competition effects of the moderately preferred items were not explicitly tested in Study 1, but items of the same stimulus class (e.g., Beanie Babies, teddy bears) were.

#### Baseline

No competing stimuli were presented. The moderately preferred item was presented with the instruction, “You can play with this.” There were no programmed consequences for target behaviors.

#### Competing stimulus continued evaluation

The experimenters presented items randomly without replacement until all items were evaluated. Target stimuli were presented with augmentations (i.e., re‐presentation, prompting, DRA, and response blocking were implemented as necessary), and generalization stimuli were presented in the absence of augmentations because they had not been presented with such procedures previously. For the DRA, Sabir earned six tokens for item engagement on a variable schedule (i.e., approximately every 30–90 s). Following token exchanges, the session was paused and resumed following reinforcer consumption. Supporting Information N summarizes the occurrence of target behavior during reinforcer consumption. Dakota accessed the iPad on a schedule similar to Sabir's (i.e., approximately every 30–90 s). No consequences were provided for item engagement or target behavior when evaluating generalization stimuli to mimic the repeated free‐access condition from the A‐CSA.

#### Participant choice

Participants were shown all four included competing stimuli and instructed to “pick one.” All other procedures were identical to the competing stimulus continued evaluation sessions. The participant choice condition ceased after participants selected one item four more times than any other (Hanratty & Hanley, 2021).

#### Generalization

The experimenters conducted generalization probes in other locations across the school (e.g., multipurpose room, game room). Generalization probes took place during the baseline and treatment phases of the study. All other procedural elements remained the same.

#### Maintenance and durability of treatment effects

Using participant choice procedures, the experimenters conducted maintenance sessions to measure the continued efficacy of the competing stimuli 1, 2, and 4 weeks following the final treatment evaluation session. Participants were exposed to the procedures and items between sessions as part of their educational goals.

### Social validity

Table 5 outlines the survey that was administered anonymously to evaluate the study's goals, procedures, and outcomes. Participants' caregivers, several BCBAs and instructors who were not associated with the study, and BCBAs and instructors who implemented the procedures completed the survey. Caregivers and both BCBAs and instructors who were not associated with the study were provided video recordings and a written description of the procedures. Respondents were allowed to select *strongly agree* (5), *agree* (4), *neutral* (3), *disagree* (2), or *strongly disagree* (1). Supporting Information O provides additional information about the survey. If respondents selected *neutral* or lower, they were asked (but not required) to indicate the reason for that rating.

**TABLE 5:** Results of social validity surveys.

Item	Information presented	Statement	*Mdn* (Range)					
			Caregiver	BCBA^1^	BCBA	Instructor^1^	Instructor	Overall
1	Purpose of the study	I think this study's goals were important to the literature on stereotypy treatment.	5 (5)	5 (5)	5 (5)	4.5 (4–5)	4 (4)	5 (4–5)
2	Participant characteristics	Decreasing stereotypy was an important goal for these participants.	5 (5)	5 (5)	5 (5)	4.5 (4–5)	4 (4)	5 (4–5)
3	Description of participants' stereotypy	It was acceptable to identify items that competed with participants' stereotypy.	5 (5)	5 (5)	5 (5)	5 (4–5)	4 (4)	5 (4–5)
4	Summary of procedures and A‐CSA flowchart	I think that using prompting, DRA, and response blocking were acceptable modifications within the assessment.	4 (4)	5 (5)	5 (5)	5 (4–5)	4.5 (4–5)	5 (4–5)
5	Summary of procedures and A‐CSA flowchart	Providing access to competing items was an acceptable behavior reduction procedure for these participants in a school setting.	5 (5)	5 (5)	4.5 (4–5)	5 (4–5)	4.67 (4–5)	5 (4–5)
6	Overview of generalization probes	I think testing whether participants engaged with multiple items for longer periods of time and across multiple settings was an appropriate way to assess generalization.	5 (5)	5 (5)	5 (5)	5 (4–5)	4 (4)	5 (4–5)
7	A‐CSA graphs & treatment graphs	I think these procedures were found to be effective.	5 (5)	5 (5)	5 (5)	4.5 (4–5)	4 (4)	5 (4–5)
8	Description of *M* stereotypy and item engagement	I would use this intervention to decrease stereotypy and increase item engagement if I had similar clients.	4 (4)	5 (5)	5 (5)	4.5 (4–5)	4.5 (4–5)	5 (4–5)
9	Description of responding during generalization probes	If I had similar clients, I would use the same procedures to assess generalization.	4 (4)	5 (5)	4.5 (4–5)	4.5 (4–5)	4 (4)	4.5 (4–5)
10	Overall findings	I would be willing to implement these procedures for similar children with ASD.	5 (5)	5 (5)	5 (5)	5 (4–5)	4 (4)	5 (4–5)

*Note*: The specific statements were altered slightly for the caregiver survey (e.g., “It was acceptable to find items that competed with my child's stereotypy”).

^1^ Indicates that the form was sent to individuals who helped implement the study procedures. A‐CSA = augmented competing stimulus assessment; ASD = autism spectrum disorder; DRA = differential reinforcement of alternative behavior; *Mdn* = Median.

Additionally, participants completed a concurrent‐chains preference assessment to assess their preference between treatment and free access to stereotypy (Hanley, 2010). The experimenter stood behind Sabir and sat across from Dakota to conduct the assessment. This was to ensure that Dakota could not mouth the cards. Colored cards that were not previously associated with study procedures were randomly assigned to the two conditions across participants, and the position of each card rotated across trials. Before implementing selection trials, participants completed 10 exposure trials (five of each condition). The experimenter prompted the selection response and conducted a brief exposure to each condition. Brief exposure lasted up to 2 min. Participants had free access to either stereotypy (no item presented) or a competing stimulus under the participant choice contingencies. If participants chose generalization stimuli, they had free access to the item; if they selected a target stimulus, they experienced response blocking contingent on target behavior and DRA for engagement with the item. During selection trials, the experimenter placed the colored cards on the table approximately 0.3 m from the participant and instructed the participant to “Pick one.” The participant then experienced the contingencies paired with their selection for 2 min. Trials were conducted until participants selected one condition in three consecutive trials.

### Results and discussion

**FIGURE 5:**
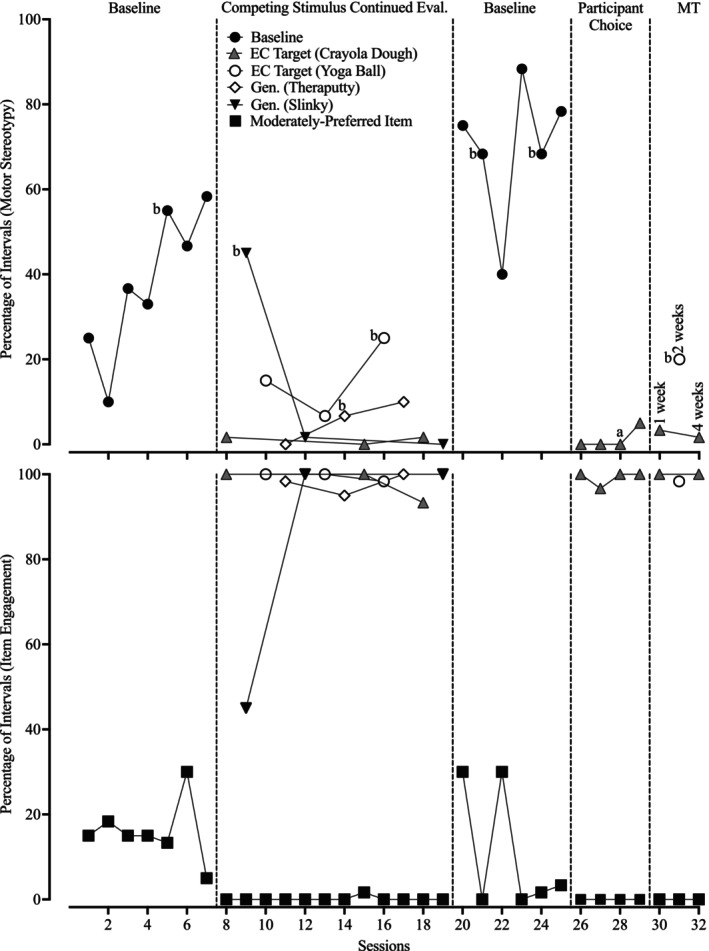
Sabir's treatment evaluation data. The lowercase letter “a” indicates that the session was conducted in an alternative classroom. The lowercase letter “b” indicates that the session was conducted in the game room. Eval = evaluation; EC = effective‐competing; Gen = generalization stimulus; Target = target stimulus; and MT = maintenance.

**FIGURE 6:**
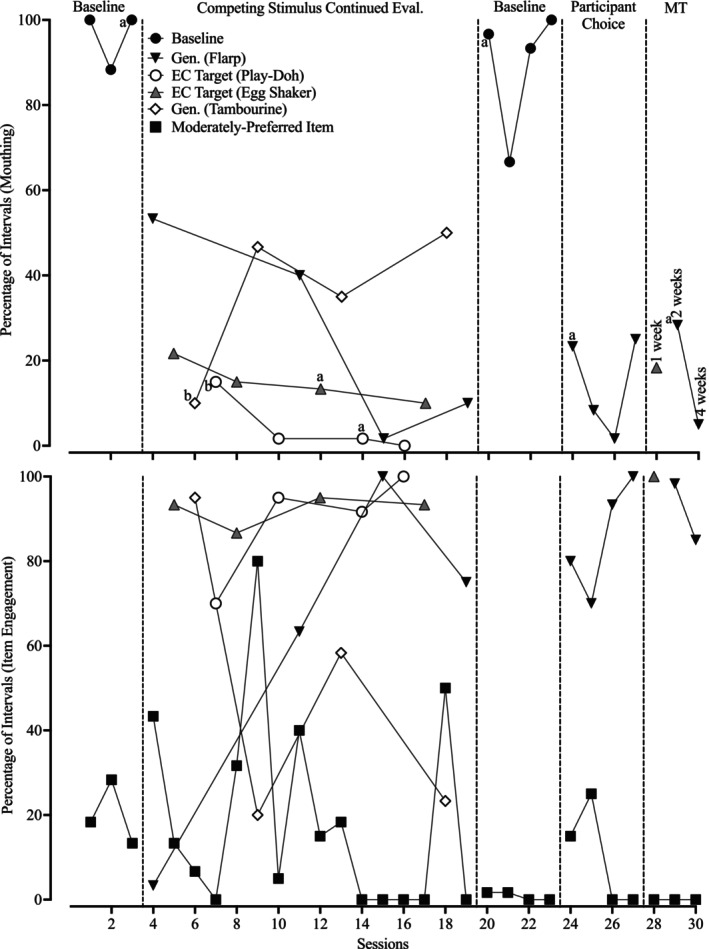
Dakota's treatment evaluation data. The lowercase letter “a” indicates that the session was conducted in an alternative classroom. The lowercase letter “b” indicates that the session was conducted in the game room. Eval = evaluation; EC = effective‐competing; Gen = generalization stimulus; Target = target stimulus; and MT = maintenance.

**FIGURE 7:**
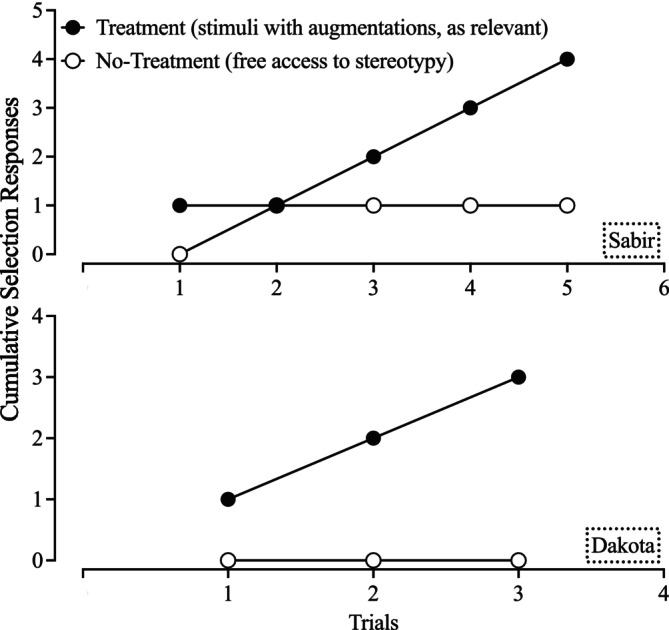
Concurrent‐chains preference assessment results.

Figures 5, 6, 7 detail the results for Study 2. The top panel of Figure 5 illustrates Sabir's motor stereotypy during the treatment evaluation; the bottom panel depicts item engagement. During the initial baseline, Sabir engaged in low to moderate levels of item engagement with the moderately preferred item as motor stereotypy increased across sessions (*M* = 37.81%, range: 10%–58.33%). Upon implementing the continued evaluation condition, item engagement was high and stable across all four items and mean motor stereotypy decreased to 9.45% (range: 0%–45%), with the most elevated data points representing generalization probes with the slinky and yoga ball. Crayola Dough (a target stimulus) produced a mean percentage of reduction of 97.06% (range: 95.58%–100%) and Theraputty (a generalization stimulus from the same stimulus class as Crayola Dough) produced a mean percentage of reduction of 85.29% (range: 82.36%–100%). Both the yoga ball (target stimulus) and slinky (generalization stimulus) produced a mean percentage of reduction of 58.85% (range for yoga ball: 33.88%–82.36%; range for slinky: −19.02%–100%). However, it is worth noting that two of the three slinky sessions resulted in large reductions in motor stereotypy. Thus, Crayola Dough and Theraputty demonstrated continued efficacy at the desired threshold. High levels of motor stereotypy and low levels of item engagement occurred during the reversal to baseline (*M* = 69.72%, range: 40%–88.33%). During participant choice, Sabir selected the Crayola Dough across four consecutive sessions; item engagement remained high, with few occurrences of motor stereotypy (*M* = 1.25%, range: 0%–5%). Across both treatment phases, access to Crayola Dough resulted in a mean percentage of reduction of 97.13% (range: 92.83%–100%). During two maintenance sessions, Sabir selected Crayola Dough and demonstrated similarly low levels of motor stereotypy (*M* = 2.50%, range: 1.67%–3.33%). When Sabir selected the yoga ball, he exhibited higher levels of motor stereotypy (i.e., 20%), consistent with previous yoga ball sessions. Figure 7 shows that Sabir selected competing stimuli in four of five trials of the concurrent‐chains preference assessment. Anecdotally, he requested competing stimuli during the trial in which he selected free access to stereotypy.

Overall, item access resulting in 80% reductions in the A‐CSA continued to yield similar results during 10‐min sessions (7 min longer than assessment trials) for Sabir. Sabir always exhibited motor stereotypy within the first minute of baseline sessions, but motor stereotypy was often delayed or entirely suppressed when he accessed competing stimuli. Despite the association of both Crayola Dough and yoga ball with reinforcement and blocking, Crayola Dough yielded more consistent reductions in motor stereotypy than yoga ball, resulting in fewer applications of response blocking. This may have occurred partly due to Sabir's apparent preference for Crayola Dough. Notably, Sabir's DRA contingency resulted in access to food. This procedure shows promise in reducing automatically maintained challenging behavior but requires further research (Rooker et al., 2022). For example, Sabir emitted motor stereotypy during reinforcer consumption and participants in Rooker et al. (2022) demonstrated decreased self‐injury when provided with food contingent on switch presses. Had Rooker et al. required more complex or effortful responses, perhaps participants' responding would have demonstrated increased variability, so further investigations into the suppressive effects of access to food without competing stimuli are warranted.

Figure 6 depicts Dakota's responding during the treatment evaluation. Mouthing occurred at high levels within the initial baseline (*M* = 96.11%, range: 88.33%–100%), and item engagement with the moderately‐preferred item was low. The mean percentage of reduction with Play‐Doh (a target stimulus) was 95.22% (range: 84.39%–100%) and 84.39% (range: 77.45%–89.59%) with the egg shaker (target stimulus). Flarp (generalization stimulus) and the tambourine (generalization stimulus) had more moderate reductions in mouthing (i.e., Flarp: 72.69%, range: 44.51%–98.26%; tambourine: 63.15%, range: 47.97%–89.59%). Therefore, the target stimuli continued to compete across settings in the school under leaner reinforcement schedules when session duration increased by 7 min. Dakota's mouthing behavior immediately increased during the reversal to baseline (*M* = 89.17%, range: 66.67%–100%). During the participant choice condition, Dakota selected Flarp across four consecutive sessions. The mean percentage of mouthing was 14.58% of intervals (range: 1.67%–25%) for a mean percentage of reduction of 83.65% (range: 71.96%–98.13%). Engagement with Flarp was consistently high. In maintenance, Dakota first selected the egg shaker and mouthing occurred in 18.33% of intervals. When she selected Flarp, item engagement was high and mouthing varied (*M* = 16.67%, range: 5%–28.33%) but remained relatively low. Figure 7 shows Dakota's cumulative selection responses. Dakota exclusively chose access to competing stimuli paired with reinforcement and response blocking during the concurrent‐chains preference assessment.

Although substantial percentages of reduction were observed, the variability of Dakota's mouthing indicates that her behavior may require more complex or choice‐based interventions. Flarp required repeated exposures to observe a mean percentage of reduction like that in the A‐CSA, and then Dakota selected it exclusively in the participant choice condition. These data suggest that A‐CSAs might predict the effectiveness of generalization stimuli, but this claim requires additional investigation. Also, evaluating generalization stimuli that were identified in the free‐access period would have allowed us to compare the effectiveness of items that were identified before and after the A‐CSA sequence. Moreover, Flarp often delayed mouthing's onset by 5 min or more as opposed to both baselines (i.e., mouthing always occurred within the first 1 min 30 s). These data suggest that engaging with Flarp produced reinforcers independently of pairing with other reinforcers (i.e., the iPad). More research on this potential phenomenon is warranted.

Table 5 outlines the results of the social validity survey. Eight of 19 surveys were returned (i.e., a 42.11% return rate). Individuals who implemented the procedures ranked the appropriateness of competing stimuli in a school setting slightly more favorably than those who did not assist with study procedures; however, all respondents (including caregivers) reported that they would implement these procedures. Additional data on the social validity of procedures to treat behaviors maintained by automatic reinforcement are needed. Although participating staff found the treatment to be socially valid, it is important to collect measures in other school settings and determine whether these procedures are seen as disruptive or valuable.

## GENERAL DISCUSSION

This study assessed whether a proposed stereotypy subtyping model aligned with an A‐CSA sequence and predicted necessary augmentations. Despite possible evidence that applying subtypes to stereotypy may have generality, the predictive validity and amenability to treatment in free‐play arrangements remain unclear. Competing stimuli were identified for all participants during the free‐access condition in the A‐CSA, and at least one generalization stimulus presented without augmentations produced an 80% reduction during the treatment evaluation. These data align with the CSA outcomes reported by Laureano et al. (2023) and Frank‐Crawford et al. (2023); however, Wunderlich et al. (2022) found that only 20% of participants with Subtype 1 stereotypy experienced positive outcomes from noncontingent reinforcement procedures alone. Furthermore, all participants in the current investigation demonstrated Subtype 1 stereotypy, limiting conclusions about the potential augmentation requirements per subtype. Preliminary evidence also suggests that augmentations within CSAs may promote generalized target behavior reductions within stimulus classes, but additional investigation is warranted.

This study replicates the extant literature by progressing through increasingly robust A‐CSA procedures to determine the necessary components for identifying competing stimuli (Hagopian et al., 2020; Jennett et al., 2011; Leif et al., 2020; Rosenzweig et al., 2024; Schmidt et al., 2021) such as response blocking for two participants. Response blocking has been a necessary component in treating many cases of behavior maintained by automatic reinforcement (Wunderlich et al., 2022) and likely functioned as a punisher in this context (i.e., it allowed us to identify competing stimuli quickly, and neither participant demonstrated target behavior reductions with the target stimuli when it was removed). Engagement with the stimuli that we assessed may or may not have produced reinforcers that were similar to those produced by target behaviors (Haddock & Hagopian 2020). For example, Sabir's engagement with Crayola Dough may have resulted in sensory consequences in his hands that were similar to those from motor stereotypy on surfaces. However, Dakota's engagement with Flarp would not produce oral sensory consequences similar to mouthing. Our findings also support claims by Ruckle et al. (2022) that A‐CSAs may identify and establish competing stimuli.

The current investigation also extends the literature in several ways. First, assessments and treatment evaluations following the stereotypy subtyping model as applied by Wunderlich et al. (2022) have yet to be published to our knowledge. Second, like Leif et al. (2020) and Rosenzweig et al. (2024), our study provides further evidence of the efficacy of A‐CSA procedures in schools. Furthermore, this study extended previous work by assessing generalization in multiple locations across the school during 10‐min sessions and included maintenance probes. Third, this study is the first to our knowledge to evaluate generalized target behavior reductions within stimulus classes following A‐CSA procedures. Fourth, we collected objective data on participants' preference for (a) different competing stimuli and (b) competing stimuli over the target response by incorporating a participant choice condition and concurrent‐chains preference assessment to obtain social validity measures from a variety of sources, including the participants.

Of course, the current study has limitations. By running a pairwise comparison during the functional analysis, other response patterns that could have informed subtyping and treatment were potentially missed (Virues‐Ortega et al., 2022). Alternative subtyping models, conducting extended‐alone screenings allowing participants free access to leisure items, or separating topographies of stereotypy into distinct classes for subtyping may yield greater predictive validity. Perhaps response classes that are maintained by automatic reinforcement with topographically diverse responses, like Sabir's, are more treatment resistant because more potential reinforcers are available; one stimulus might not compete with all the potential reinforcers. If there was such differentiation, separating these responses for subtyping could have highlighted which responses needed particular levels of augmentation.

Similarly, the items present during the functional analysis control sessions could have influenced the subtyping results; for example, Sabir's control included a yoga ball, which later demonstrated a competition effect in the A‐CSA. We might have observed a different pattern of responding in the functional analysis using another item. Dakota's results also suggest that the presence of a less‐preferred item for mouthing likely plays a role in subtyping and overall responding within functional analyses. As noted by Hagopian et al. (2017) and Wunderlich et al. (2022), visual analysis of functional analysis data is an integral part of subtyping procedures and has resulted in modifications to subtyping guidelines to account for observed level changes and outlier data points, among other things. Dakota's functional analysis data support the need for detailed guidelines that are informed by visual analysis when subtyping stereotypic behavior.

Although we developed stimulus classes with high levels of interobserver agreement, defining the parameters of stimulus classes can be challenging and we did not control the number of hypothesis‐based and non‐hypothesis‐based stimuli per class. Furthermore, the current study used stimulus classes that were developed by the first author rather than via group consensus. Future researchers should have a second, independent experimenter complete the same activity for developing stimulus classes and obtain agreement prior to implementation. This person could also complete interobserver agreement for critical and noncritical features.

Additionally, participants completed the A‐CSA despite identifying competing stimuli in the free‐access condition given our objective of evaluating potential generalized effects. Future researchers could bypass augmentation trials with target stimuli after identifying competing generalization stimuli during the free‐access condition and evaluate them in treatment evaluations or compare the efficacy of items identified in the free‐access trials with those in the repeated free‐access trials. Similarly, we did not account for response variability when identifying and validating competing stimuli, which future researchers should consider. The current study presents data that are averaged across trials per item, preventing an evaluation of trends per item. Moreover, some of the generalization stimuli identified as competing stimuli in the free‐access condition were not identified as competing stimuli in the repeated free‐access condition. Plus, some competing generalization stimuli were from different stimulus classes than those of the competing target stimuli, so it is not clear whether augmenting tactics produced generalization within or across stimulus classes. The observed variability of target behaviors also makes it unclear whether generalization of competition effects even occurred. Future research that replicates these procedures with limited items in a reversal design to better analyze session‐by‐session data can address these limitations. Conducting additional generalization stimulus trials and evaluating more potential competing stimuli might help to elucidate whether the reductions in target behavior resulted from multiple‐exemplar training (through augmenting target stimuli and presenting generalization stimuli of the same stimulus classes) or response variability.

Some of the competing stimuli required augmentation during treatment, so it remains unclear how participants would have responded had we removed those tactics during the treatment evaluation. Despite not specifically scoring each implementation of blocking, decreased levels of target behavior resulted in fewer implementations. However, developing augmentation‐fading procedures would be beneficial. Also, Dakota's selection of Flarp over more effective stimuli raises interesting ethical considerations. When highly preferred items do not yield sufficient reductions in target behavior, perhaps practitioners can accommodate both efficacy and preference in treatment by assessing moderately preferred items for competition effects and incorporating stimulus rotation into free‐access protocols. Future researchers could also consider measuring target behavior and item engagement during preference assessments involving longer access periods with items. Then, researchers can design control sessions within functional analyses that include either a high‐preferred and high‐competing stimulus or a high‐preferred and low‐competing stimulus to better explain potential causes for observed response patterns during the assessment.

We encourage future researchers to extend this line of research by evaluating the best arrangements for competing stimuli in settings where prolonged access to leisure items may be counterproductive. Noncontingent access to competing stimuli may be socially valid in some settings, but measures of treatment practicality are warranted. Much like Leif et al. (2020) suggested, it could be beneficial to assess whether competing stimuli develop a reinforcing function over time; by extension, future researchers should measure the continued need for, or removal of, response blocking in these arrangements. Obtaining quantitative measures of the item efficacy when caretakers are absent would help determine whether response blocking could be removed from treatment. Competing stimuli may bolster environmental enrichment procedures and mitigate relapse when other treatment components encounter disruptions, too (Ruckle et al., 2022). Additional research into factors that could make some response classes of behavior maintained by automatic reinforcement more resistant to treatment (e.g., varied topographies within the response class) is warranted (Lory et al., 2023). Analyzing participant data at the molecular level for response patterns with competing stimuli may yield information on the necessity of stimulus rotation as well.

Last, future researchers could develop decision‐making frameworks with respect to A‐CSAs. Investigations into treatments using competing stimuli following the use of decision‐making frameworks are warranted given that the CSA itself may not require sophisticated training. Such investigations should consider conducting caregiver training in CSAs and measuring the caregiver's selection of competing stimuli. Enabling caregivers to intervene on behaviors that are maintained by automatic reinforcement without extensive training may prove highly motivating and socially valid to some participants. Similarly, future studies may be designed to determine whether specific stimulus characteristics can be identified, shared with caregivers, and inform additional competing stimulus selection.

The current study combined the literature on stereotypy subtyping and A‐CSA sequences. A‐CSAs have been documented to effectively identify more competing stimuli than traditional CSAs, so it may be beneficial to manualize A‐CSA procedures for researchers and practitioners alike. This study helps bring the field closer to this objective and highlights previously uninvestigated effects (i.e., generalization) of A‐CSAs.

## AUTHOR CONTRIBUTIONS

Samantha Breeman designed the study with direct oversight from Jason Vladescu. Tina Sidener, Ruth DeBar, and Danielle Gureghian provided feedback on the study design. Samantha Breeman and Jason Vladescu obtained IRB approval. Samantha Breeman managed recruitment. Samantha Breeman conducted select study sessions, and collected and summarized data. Samantha Breeman analyzed the results with Jason Vladescu. Samantha Breeman wrote the first draft of the manuscript with feedback from Jason Vladescu. All authors revised and approved the final manuscript.

## CONFLICT OF INTEREST STATEMENT

The first and fifth authors were affiliated with the private school where participants were recruited during the study. All other authors declare they have no conflicts of interest.

## ETHICS APPROVAL

This study was approved by the human subjects' institutional review board at Caldwell University. The study was performed in accordance with the ethical standards as laid down in the 1964 Declaration of Helsinki and its later amendments or comparable ethical standards.

## Supporting information


**Data S1:** Supporting Information

## Data Availability

The data that support the findings of this study are available from the corresponding author upon reasonable request.
