# Supplementary material for: Generalized target behavior reductions and maintenance of effects following an augmented competing stimulus assessment sequence
Source: J Appl Behav Anal. 2025 Jun 18;58(3):573–94. doi: 10.1002/jaba.70021 (PMC12302334; doi:10.1002/jaba.70021)
Supplement: Supplementary file 1 — Data S1: Supporting Information [file JABA-58-573-s001.pdf]

Supporting Information

Supporting Information A

Overview of Previous A-CSA Conditions

Citation	A-CSA Conditions Included									
	**No-Stimulus Control**	**Free Access/No Prompting**	**P**	**P+RB**	**RB**	**Re**	**Re + RB**	**P + PE**	**P + DRA**	**Repeated Free Access**	**Total Stimuli Assessed**
Falligant, Carver et al. (2021)	X				X						4
Falligant, Hardesty et al. (2021)	X							X			6
Frank-Crawford et al. (2023)	X	X	X	X						X	5–2
Hagopian et al. (2020)	X	X	X	X						X	7–16
Jennett et al. (2011)	X	X				X	X				16
Leif et al. (2020)	X	X	X						X		7–8
Rosenzweig et al. (2024)	X	X	X	X						X	6–12
Ruckle et al. (2022)	X	X		X						X	7
Schmidt et al. (2020)	X	X	X	X							14
Shawler et al. (2023)	X	X	X								8
Thomas et al. (2023)	X	X	X	X						X	5

Note: A-CSA = augmented competing stimulus assessment; Re = Re-presentation; P = prompted engagement; PE = protective equipment; RB = response blocking; DRA = differential reinforcement of alternative behavior. For studies with multiple participants, the range of total number of stimuli assessed is provided.

References

Falligant, J. M., Carver, A., Zarcone, J., & Schmidt, J. D. (2021). Assessment and treatment of public disrobing using noncontingent reinforcement and competing stimuli. *Behavior Analysis: Research and Practice*, *21*(1), 75–83. https://dx.doi.org/10.1037/bar0000179

Falligant, J. M., Hardesty, S. L., Pierce, D., & Kurtz, P. F. (2021). Assessment and treatment of tracheostomy tube manipulation: Effects of competing stimuli and protective equipment. *Journal of Applied Behavior Analysis*, *54*(4), 1625–1638. https://doi.org/10.1002/jaba.846

Shawler, L. A., Clayborne, J. C., & O'Connor, J. T. (2023). A competing stimuli intervention package to treat automatically reinforced destructive behavior. *Clinical Case Studies*, *22*(2), 138–154. https://doi.org/10.1177/15346501221099218

Thomas, B. R., Bowman, M. D., Sanchez, A., & Strohmeier, C. W. (2023). Parent treatment of complex pica in a teen with autism. *Behavioral Interventions*, *38*(3), 611–624. https://doi.org/10.1002/bin.1956

Supporting Information B

Assent Procedures Across Participants

**Participant**	**Method of Assent**
Dakota	Enters room without physical assistance, absence of challenging behavior (i.e., crying, SIB), or attempts to leave
Sabir	Vocal "yes" and enters room without physical assistance, absence of challenging behavior (i.e., disruption, elopement out of the room, SIB paired with crying), or attempts to leave
Sawyer	Head nod or vocal "yes" and enters room without physical assistance, absence of challenging behavior (i.e., SIB paired with crying), or attempts to leave

Note: SIB = self-injurious behavior.

Supporting Information C

Additional Information about Participants and Functional Behavior Assessment

Additional Information Regarding Participants' Assessments

Participant performance on the following assessments was also included (as available): subsections of the Verbal Behavior Milestones Assessment and Placement Program (Sundberg, 2008); subsections of the Assessment of Functional Living Skills (Partington & Mueller, 2012); Gilliam Autism Rating Scale-Third Edition (Gilliam, 2013); and the Vineland Adaptive Behavior Scales-Third Edition (Sparrow et al., 2016).

Functional Behavior Assessment

Prior to the functional analysis, we conducted direct observation of the target behaviors and collected data on the antecedents and consequences surrounding target behaviors in the participant's classrooms. We also conducted a Functional Analysis Screening Tool (Iwata & DeLeon, 2005) with the participant's parents and instructional staff to rule out potential social functions for target behaviors. In the event that target behaviors appeared to occur across multiple antecedent events and consequences throughout the participants' school day, we proceeded with the functional analysis.

References

Gilliam, J. E. (2013). *Gilliam Autism Rating Scale* (3rd ed.). Austin, TX: Pro-Ed.

Iwata, B. A., & DeLeon, I. (2005). *The functional analysis screening tool.* Gainesville, FL: The Florida Center on Self-Injury, University of Florida.

Partington, J. W., & Mueller, M. M. (2012). *The Assessment of Functional Living Skills.* Walnut Creek, CA: Behavior Analysts, Inc. and Stimulus Publications.

Sparrow, S. S., Saulnier, C. A., Cicchetti, D. V., & Doll, E. A. (2016). *Vineland-3: Vineland*

adaptive behavior scales (3rd ed.). Minneapolis, MN: Pearson Assessments.

Sundberg, M. L. (2008). *Verbal Behavior Milestones Assessment and Placement Program: The VP-MAPP.* Concord, CA: AVB Press.

Supporting Information D

Indirect Assessment Results Across Participants

Participant	VB-MAPP Subsections					AFLS Home Skills: Leisure	GARS–3 ASD Index	Vineland–3 ABC Score
	Motor Imitation	Independent Play	Mands	Social Play	Barriers			
Dakota	3.50	2.50	5	3	38	20	97	20
Sabir	9.50	10	10.50	8	39	16	92	20
Sawyer	8.50	9.50	4	2.50	31	8	103	27

Note: Results of the Verbal Behavior Milestones Assessment and Placement Program (VB-MAPP) and Assessment of Functional Living Skills (AFLS) are presented as total accumulated points per subsection. The Gilliam Autism Rating Scale (Third Edition) standardized autism index are shown above. The Vineland (Third Edition) adaptive behavior composite (shown above) provides an overall measure of adaptive behavior across multiple domains. ASD = autism spectrum disorder

Supporting Information E

Functional Analysis Procedural Fidelity Data Sheets

No-Interaction Condition	Behavior	Score
	Timer set for 10 min	
	Tells participant, "I have to work. You play here."	
	Closes door	
	No programmed consequences delivered	
	Total Correct/Total Prescribed	_______/_______ x 100 =

Control Condition	Behavior	Score
	Timer set for 10 min	
	Room has highly-preferred items/snacks available	
	Tells participant, "Let's play!"	
	Allows participant free access	
	Provides praise/attn every 30 s; 5 s delay if target occurs at time	
	No consequence for target behavior	
	Total Correct/Total Prescribed	_______/_______ x 100 =

Supporting Information F

A-CSA Conditions Procedural Fidelity Data Sheets

Control Condition	Behavior	Score
	Timer set for X	
	No item presented	
	No consequences for target behavior	
	Total Correct/Total Prescribed	_______/_______ x 100 =

Free Access/ Repeated Free Access/ Generalization Condition	Behavior	Score
	Timer set for X	
	Places item within arm's reach	
	Says, "You can play with this if you want"	
	No consequences for target behavior	
	Total Correct/Total Prescribed	_______/_______ x 100 =

Re-presentation Condition	Behavior	Score
	Timer set for X	
	Places item within arm's reach	
	Says, "You can play with this if you want"	
	Repeats "You can play with this if you want" following 10 s no engagement	
	Total Correct/Total Prescribed	_______/_______ x 100 =

Re-presentation and Prompting Condition	Behavior	Score
	Timer set for X	
	Places item within arm's reach	

	Says, "You can play with this if you want" and prompts engagement (3 s max)	
	Discontinues prompt if participant resists	
	Total Correct/Total Prescribed	_______/_______ x 100 =

**Re-presentation, Prompting, & DRA Condition**	**Behavior**	**Score**
	Timer set for X	
	Places item within arm's reach	
	Says, "You can play with this if you want" and prompts engagement (3 s max)	
	Delivers reinforcer following 10 s engagement	
	Discontinues prompt if participant resists	
	Total Correct/Total Prescribed	_______/_______ x 100 =

**Re-presentation, Prompting, DRA, & Blocking Condition**	**Behavior**	**Score**
	Timer set for X	
	Places item within arm's reach	
	Says, "You can play with this if you want" and prompts engagement (3 s max)	
	Delivers reinforcer following 10 s engagement	
	Discontinues prompt if participant resists	
	Blocks target behavior (3 s)	
	Total Correct/Total Prescribed	_______/_______ x 100 =

Note: A-CSA = augmented competing stimulus assessment; DRA = differential reinforcement of alternative behavior.

Supporting Information G

Hypothesis-Based Stimuli for Sabir

Target Stimuli	Hypothesis-Based Target Stimuli	Generalization Stimuli (n of Noncritical Feature Variations)	Hypothesis-Based Generalization Stimuli
Tambourine<br><br>Sequin Dinosaur<br><br>Yoga Ball[1]<br><br>Kaleidoscope<br><br>Crayola Dough	3 (tactile sensation in hands: tambourine, sequin dinosaur, Crayola Dough)<br><br>2 (auditory sensation: tambourine, yoga ball)<br><br>1 (tactile sensation in body: yoga ball)	Play Doh (3)<br>Theraputty (4)<br>Elmer's Slime (3)<br>Flarp (4)<br>Butter Slime (4)<br>Teddy Bear (5)<br>Pillow Pet (5)<br>Hatchimal (4)<br>Beanie Baby (5)<br>Emoji Pillow (4)<br>View Finder (5)<br>Liquid Drop Tube (5)<br>Bubble Blower (5)<br>Magna Doodle (5)<br>Book (5)<br>Stress Ball (4)<br>Slinky (5)<br>Expandable Ball (4)<br>Bouncy Ball (4)<br>Pin Toy (5)<br>Rain Stick (5)<br>Maraca (5)<br>Bells (5)<br>Hand Clapper (5)<br>Egg Shaker (5)	14 (tactile sensation in hands: Play Doh, Theraputty, Elmer's Slime, Flarp, Butter Slime, teddy bear, Pillow Pet, Hatchimal, Beanie Baby, emoji pillow, stress ball, liquid drop tube, expandable ball, pin toy)<br><br>8 (auditory sensation: rain stick, maracas, bells, hand clapper, egg shaker, pin toy, slinky, Magna Doodle)<br><br>2 (tactile sensation in body: expandable ball, bouncy ball)

Note: Designations of stimuli as hypothesis-based and non-hypothesis based were determined by the first author following observation of the target behaviors and functional play with the included stimuli. As such, all designations and proposed reinforcers are speculative, and potential reinforcers for engaging with included stimuli in other ways were not listed. We also elected to use the terms “hypothesis-based” and “non-hypothesis based” as this more accurately reflects the relation between the potential stimulus reinforcers (i.e., we did not specifically test for matching, so the potential reinforcers are based on our hypothesis for those of the target behaviors). A “[1]” indicates the stimulus was also present during the control sessions of the functional analysis.

Supporting Information H

Hypothesis-Based Stimuli for Sawyer

Target Stimuli	Hypothesis-Based Target Stimuli	Generalization Stimuli (n of Noncritical Feature Variations)	Hypothesis-Based Generalization Stimuli
Flarp<br><br>Pillow Pet<br><br>Bubble Blower<br><br>Pin Toy<br><br>Hand Clapper	3 (visual sensation: bubble blower, hand clapper, pin toy)<br><br>4 (tactile sensation in hands: Flarp, Pillow Pet, pin toy, hand clapper)	Play Doh (3)<br>Theraputty (4)<br>Elmer's Slime (3)<br>Crayola Dough (4)<br>Butter Slime (4)<br>Teddy Bear (5)<br>Sequin Dinosaur (5)<br>Hatchimal (4)<br>Beanie Baby (5)<br>Emoji Pillow (4)<br>View Finder (5)<br>Liquid Drop Tube (5)<br>Kaleidoscope (5)<br>Magna Doodle (5)<br>Book (5)<br>Stress Ball (4)<br>Slinky (5)<br>Expandable Ball (4)<br>Bouncy Ball (4)<br>Yoga Ball (5)<br>Rain Stick (5)<br>Maraca (5)<br>Bells (5)<br>Tambourine (5)<br>Egg Shaker (5)	7 (visual sensation: kaleidoscope, Magna Doodle, book, slinky, liquid drop tube, expandable ball, View Finder)<br><br>11 (tactile sensation in hands: Play Doh, Theraputty, Elmer's Slime, Crayola Dough, butter slime, teddy bear, sequin dinosaur, Hatchimal, emoji pillow, stress ball, expandable ball)

Note: Designations of stimuli as hypothesis-based and non-hypothesis based were determined by the first author following observation of the target behaviors and functional play with the included stimuli. As such, all designations and proposed reinforcers are speculative, and potential reinforcers for engaging with included stimuli in other ways were not listed. We also elected to use the terms "hypothesis-based" and "non-hypothesis based" as this more accurately reflects the relation between the potential stimulus reinforcers (i.e., we did not specifically test for matching, so the potential reinforcers are based on our hypothesis for those of the target behaviors).

Supporting Information I

Hypothesis-Based Stimuli for Dakota

Target Stimuli	Hypothesis-Based Target Stimuli	Generalization Stimuli (n of Noncritical Feature Variations)	Hypothesis-Based Generalization Stimuli
Play-Doh	0 (tactile sensation in mouth)	Crayola Dough (3)	0 (tactile sensation in mouth)
		Theraputty (4)	
Emoji Pillow	5 (general tactile sensation: Play Doh, emoji pillow, liquid drop tube, expandable ball, egg shaker)	Elmer’s Slime (3)	15 (general tactile sensation: Crayola Dough, Theraputty, Elmer’s Slime, Flarp, butter slime, teddy bear, Pillow Pet, Hatchimal, Beanie Baby, sequin dinosaur, stress ball, slinky, yoga ball, bouncy ball, pin toy)
Liquid Drop Tube		Flarp (4)	
		Butter Slime (4)	
Expandable Ball		Teddy Bear (5)	
		Pillow Pet (5)	
Egg Shaker		Hatchimal (4)	
		Beanie Baby (5)	
		Sequin Dinosaur (4)	
		View Finder (5)	
		Kaleidoscope (5)	
		Bubble Blower (5)	
		Magna Doodle (5)	
		Book (5)	
		Stress Ball (4)	
		Slinky (5)	
		Yoga Ball (4)	
		Bouncy Ball (4)	
		Pin Toy (5)	
		Rain Stick (5)	
		Maraca (5)	
		Bells (5)	
		Hand Clapper (5)	
		Tambourine (5)	

Note: Designations of stimuli as hypothesis-based and non-hypothesis based were determined by the first author following observation of the target behaviors and functional play with the included stimuli. As such, all designations and proposed reinforcers are speculative, and potential reinforcers for engaging with included stimuli in other ways were not listed. We also elected to use the terms “hypothesis-based” and “non-hypothesis based” as this more accurately reflects the relation between the potential stimulus reinforcers (i.e., we did not specifically test for matching, so the potential reinforcers are based on our hypothesis for those of the target behaviors).

Supporting Information J

Stimulus Classes, Members, Critical, and Non-critical Features

Stimulus Class	Class Members	Critical Features	Non-critical Features Varied
Malleable Shape Toys	Play-Doh<br>Crayola Dough<br>Theraputty<br>Elmer's Slime<br>Flarp<br>Butter Slime	Malleable/Shape changes and does not remain intact<br>Maintains malleability across uses	Color of toy and container<br>Size of container<br>Firmness of toy<br>(4 variations)
Plush Touch Toys	Sequin Dinosaur<br>Teddy Bear<br>Pillow Pet<br>Hatchimal<br>Beanie Baby<br>Emoji Pillow	Soft texture<br>Fabric exterior with stuffed interior	Shape of toy<br>Color of fabric<br>Size of toy<br>Ability to pose limbs<br>Exterior fabric material<br>(5 variations)
Visual Effects Toys	Kaleidoscope<br>View Finder<br>Liquid Drop Tube<br>Bubble Blower<br>Magna Doodle<br>Book	Engagement produces a visual stimulus previously not visible	Shape of toy<br>Size of toy<br>Color of toy<br>Visual stimulus produced<br>Toy material<br>How visual is produced<br>(6 variations)
Kinesthetic Tactile Toys	Stress Ball<br>Slinky<br>Expandable Ball<br>Yoga Ball<br>Bouncy Ball<br>Pin Toy	Frame/Shape moves but remains intact	Shape of toy<br>Size of toy<br>Color of toy<br>Direction of intended motion<br>Product material<br>(5 variations)
Shaken Noise-Maker Toys	Rain Stick<br>Tambourine<br>Maraca<br>Bells<br>Hand Clapper<br>Egg Shaker	Auditory stimulus results from item components colliding	Shape of toy<br>Size of toy<br>Color of toy<br>Auditory stimulus produced by toy<br>Product material<br>(5 variations)

Note: All defined critical and non-critical features were defined by the first author based on possible variations that would not alter the potential reinforcers for functional play with included items.

Supporting Information K

Additional Information about the Reinforcer Assessment

Additional Information Regarding the Reinforcer Assessment

We conducted a concurrent-operant reinforcer assessment (Piazza et al., 1996) with all participants. The purpose of conducting this assessment was to ensure that we provided proven reinforcers during the differential reinforcement of alternative behaviors (DRA) component of the augmented competing stimulus assessment (A-CSA, if necessary). We assessed praise and tokens separately as compared to a control (i.e., one response option resulted in the consequence being evaluated, the other did not produce any consequences). For Sabir, target responses were defined as placing blocks into one of two bins. For Dakota, target responses were defined as touching one of two magnetic tiles. Sawyer's target responses were defined as touching one of two images. Session duration was also measured in seconds. Data were summarized each session as rate (per min). The session duration in seconds was divided by 60; then, the frequency of responses was divided by the previous quotient.

Sabir's mean rate of responding to praise was 5.50 responses per min (range: 5.13–5.83) as opposed to 1.83 responses per min (range: 1.71–1.94) for extinction; his mean rate of responding to tokens was 8.96 responses per min (range: 7.69–9.61) as compared to 3.42 responses per min (range: 1.59–4.84) for extinction. Thus, both praise and tokens appear to function as reinforcers, with tokens being more potent. Dakota's responding to praise ($M = 0.88$ responses per min, range: 0–2.33) and tokens ($M = 0$ responses per min) did not differ significantly from her responding to extinction ($M = 0.54$ responses per min, range: 0–1.64), so we assessed whether access to her iPad functioned as a reinforcer. Her mean rate of responding immediately increased ($M = 7.55$ responses per min, range: 6.33–9.33) and differentiation was

observed (i.e., extinction *M* = 2.22 responses per min, range: 1.33–3.67). Sawyer's mean rate of responding for tokens was 3.76 responses per min (range: 3–4.29) as compared to 0 for extinction. His mean rate of responding for praise was similar (*M* = 3.51 responses per min, range 2.34–4.29), whereas mean rate of responding for extinction remained low (*M* = 0.74, range 0–1.43).

References

Piazza, C. C., Fisher, W. W., Hagopian, L. P., Bowman, L. G., & Toole, L. (1996). Using a choice assessment to predict reinforcer effectiveness. *Journal of Applied Behavior Analysis*, *29*(1), 1–9. https://doi.org/10.1901/jaba.1996.29-1

Supporting Information L

Percentage of Reinforcer Consumption Periods with Target Behavior during the A-CSA

**Stimulus**	**Re-presentation + Prompting + DRA**		**Re-presentation + Prompting + DRA + Response Blocking**	
	**Mean**	**Range**	**Mean**	**Range**
Expandable Ball	89.13%	76.47%–100%	28.79%	17.64%– 50%
Egg Shaker	48.91%	41.18%–55.56%	21.35%	11.11%–29.41%
Liquid Drop Tube	75.68%	68.75%–87.5%	39.83%	31.25%–47.05%
Pillow Pet	74.54%	61.11%–100%	0%	–
Play-Doh	73.53%	62.5%–86.67%	21.94%	17.65%–29.41%
Grand Mean	72.36%		22.38%	

Note: These data are only available for Dakota as Sabir did not consume his reinforcers during A-CSA trials (i.e., all snacks were eaten following A-CSA trials, so trials were not paused for reinforcer consumption). Target behavior occurred with either the iPad or other stimuli in the environment, not the target stimuli, as they were briefly removed for reinforcer consumption. A-CSA = augmented competing stimulus assessment; DRA = differential reinforcement of alternative behavior.

Supporting Information M

Treatment Evaluation Procedural Fidelity Data Sheets

Baseline Condition	Behavior	Score
	Timer set for 10 min	
	No identified competing item presented	
	Moderately-preferred item within reach	
	No consequences for target behavior	
	Total Correct/Total Prescribed	_______/_______ x 100 =

Continued Evaluation Condition	Behavior	Score
	Timer set for 10 min	
	Places item within arm's reach	
	Says, "You can play with this if you want."	
	Implements procedures from successful A-CSA condition	
	Total Correct/Total Prescribed	_______/_______ x 100 =

Participant Choice Condition	Behavior	Score
	Timer set for 10 min	
	Places all identified items within arm's reach	
	Says, "Pick one."	
	Implements procedures from successful A-CSA condition	
	Total Correct/Total Prescribed	_______/_______ x 100 =

**Generalization Condition**	**Behavior**	**Score**
	Timer set for 10 min	
	Conducts session outside of primary classroom	
	Places item within arm's reach	
	Says, "You can play with this if you want."	
	Implements procedures from successful A-CSA condition	
	Total Correct/Total Prescribed	_______/_______ x 100 =

**Maintenance Condition**	**Behavior**	**Score**
	Timer set for 10 min	
	Places item within arm's reach	
	Says, "You can play with this if you want."	
	Implements procedures from successful A-CSA condition	
	Total Correct/Total Prescribed	_______/_______ x 100 =

Note: A-CSA = augmented competing stimulus assessment.

Supporting Information N

Percentage of Reinforcer Consumption Periods with Target Behavior during Study 2

Sabir			Dakota		
Stimulus	Mean	Range	Stimulus	Mean	Range
Crayola Dough	33.33%	0%–100%	Play-Doh	17.05%	0%–50%
Yoga Ball	58.25%	0%–100%	Egg Shaker	26.15%	0%–50%
Grand Mean	41%		Grand Mean	21.60%	

Note. Data are only available for the stimuli which were presented with augmentations during treatment evaluation. The session timer was paused for reinforcer consumption. Target behavior occurred with either the reinforcer or other stimuli in the environment, not the competing stimuli, as they were briefly removed for reinforcer consumption.

Supporting Information O

Additional Information about the Social Validity Survey

Additional Information Regarding Social Validity Survey

We asked questions pertaining to the validity of these procedures in settings where skill acquisition remains an important clinical focus, and whether the outcomes demonstrated in the study are meaningful for those settings. Each survey was slightly modified and analyzed separately to account for these differences. The survey was administered via Google Forms and included descriptions of different aspects of the study and graphs for respondents to rate based on acceptability. The survey began by thanking the respondent for their participation and informing them that their name/email would not be associated with their responses. There were 10 total items, designed to assess the acceptability of the goals (Items 1–3), procedures (Items 4–6), outcomes (Items 7–9), and overall (Item 10); each item began with information about an aspect of the study (i.e., description, information graphic, or graph), followed by, “Based on this information, please rate your agreement with the following statement: (statement).”
